# Circadian Rhythms of Sense and Antisense Transcription in Sugarcane, a Highly Polyploid Crop

**DOI:** 10.1371/journal.pone.0071847

**Published:** 2013-08-06

**Authors:** Carlos Takeshi Hotta, Milton Yutaka Nishiyama, Glaucia Mendes Souza

**Affiliations:** Departamento de Bioquímica, Instituto de Química, Universidade de São Paulo, São Paulo, Brazil; University of Massachusetts Amherst, United States of America

## Abstract

Commercial sugarcane (*Saccharum* hybrid) is a highly polyploid and aneuploid grass that stores large amounts of sucrose in its stem. We have measured circadian rhythms of sense and antisense transcription in a commercial cultivar (RB855453) using a custom oligoarray with 14,521 probes that hybridize to sense transcripts (SS) and 7,380 probes that hybridize to antisense transcripts (AS).We estimated that 32% of SS probes and 22% AS probes were rhythmic. This is a higher proportion of rhythmic probes than the usually found in similar experiments in other plant species. Orthologs and inparalogs of Arabidopsis thaliana, sugarcane, rice, maize and sorghum were grouped in ortholog clusters. When ortholog clusters were used to compare probes among different datasets, sugarcane also showed a higher proportion of rhythmic elements than the other species. Thus, it is possible that a higher proportion of transcripts are regulated by the sugarcane circadian clock. Thirty-six percent of the identified AS/SS pairs had significant correlated time courses and 64% had uncorrelated expression patterns. The clustering of transcripts with similar function, the anticipation of daily environmental changes and the temporal compartmentation of metabolic processes were some properties identified in the circadian sugarcane transcriptome. During the day, there was a dominance of transcripts associated with photosynthesis and carbohydrate metabolism, including sucrose and starch synthesis. During the night, there was dominance of transcripts associated with genetic processing, such as histone regulation and RNA polymerase, ribosome and protein synthesis. Finally, the circadian clock also regulated hormone signalling pathways: a large proportion of auxin and ABA signalling components were regulated by the circadian clock in an unusual biphasic distribution.

## Introduction

The circadian clock is a signalling network that provides organisms with an endogenous timekeeping mechanism. This mechanism allows the organisms to organize their metabolism in time; to anticipate rhythmic environmental changes; to measure the length of the light and dark phases of the day; and to modulate internal and external signals according to its temporal context, a phenomenon called gating [1]. Plants with a circadian clock period that is similar to the period of environmental rhythms fix more carbon and have higher water use efficiency than plants with circadian periods that do not match with the environment [2].

The circadian clock can be divided in three different parts: the central oscillator; the input pathways and the output pathways. The input pathways, primarily regulated by light and temperature, bring environmental information to the central oscillator. Phytochromes and cryptochromes are the main photoreceptors involved in the regulation of the central oscillator. Little is known about the role of temperature in the circadian clock entrainment. The central oscillator generates the endogenous rhythms. A recent model suggested that a repressilator circuit composed of multiple transcriptional feedback loops is found in the core of the central oscillator [3]. In this model, expression of *CIRCADIAN CLOCK ASSOCIATED 1* (*CCA1*) and *LATE ELONGATED HYPOCOTYL* (*LHY)* are inhibited by the PSEUDO-RESPONSE REGULATOR 5, 7 and 9 (PRR5, PRR7 and PRR9), at the same time that CCA1 and LHY are inhibitors of the evening complex (EC) [3]. The EC is a protein complex made of LUX ARRHYTHMO, EARLY FLOWERING 3 (ELF3) and ELF4 [4]–[7]. The EC is auto-inhibited, completing a loop that was previously called the evening loop. It also inhibits the *PRRs*, which are activated by CCA1/LHY, as part of the previously called the morning loop [3]. Another component, TIME OF CHLOROPHYLL A/B BINDING PROTEIN 2 (TOC1), was considered part of the central loop through the activation of *CCA1/LHY* but there is evidence that it actually acts as an expression inhibitor [8], [9]. Other important components of the circadian clock are CCA1 HIKING EXPEDITION (CHE), GIGANTEA (GI) and ZEITLUPE (ZTL) [10]. The structure of the plant circadian clock has mostly been studied in the dicot Arabidopsis, and while most components of the circadian clock are conserved among plants, they may have different functions [11]. The output pathways take the temporal information generated from the central oscillator to regulate many physiological processes, such as photosynthesis, stomata movements and organ growth. These processes are regulated through many mechanisms: control of chromatin structure, changes in mRNA and protein stability, alternative splicing, and regulation of transcript levels [12]–[15].

A large proportion of the plant transcriptome is subjected to circadian control. Experiments using microarrays showed that 6% to 15% of the Arabidopsis, poplar, maize and rice transcriptomes are rhythmic under constant environmental conditions [13], [16]–[20]. If plants are under a cycling environment – light/dark cycles, warm/cold cycles or both – the proportion of rhythmic transcripts increases to 30% to 50% [17], [19], [21]. When all experimental setups are considered, close to 90% of all detectable Arabidopsis transcripts are rhythmic in one or more conditions [19].

Sugarcane is a C4 monocot that stores sucrose in its stem. Commercial sugarcane varieties are the result of multiple interspecific hybridizations between *Saccharum officinarum* and *S. spontaneum*, which resulted in a highly polyploid and aneuploid genome [22], [23]. The high level of sucrose accumulated in sugarcane, together with its yield, make this crop an important bioenergy feedstock in a world concerned about alternatives to fossil-based fuels [24]. Even though there is evidence that sugarcane has not reached its potential yield limit, yearly increases in sugarcane yield are low and may be plateauing, a trend that may be reversed by the introduction of new biotechnological tools [24], [25].

One strategy to develop new biotechnological tools to use in sugarcane improvement is to know more about its genome, gene networks and physiology [26]. Microarrays have been a successful strategy to identify genes of interest in sugarcane [27], [28]. We have recently developed a new custom oligoarray with more than twenty-one thousand elements with probes that hybridize to sense and antisense sugarcane EST sequences [29]. This array identified 928 differentially expressed probes in the sense direction (SS) and 59 in the antisense direction in sugarcane subjected to water suppression [29], adding considerable knowledge to previous experiments that hybridized the same samples using a 1,545 elements microarray and identified 93 differentially expressed transcripts probes [27].

Natural antisense transcripts (NATs) have been shown to regulate transcription, processing and degradation of their sense cognate [30]–[33]. For example, *AtCOOLAIR*, a cold-induced NAT, has been associated with transcriptional silencing of its cognate *AtFLOWERING LOCUS C* (*AtFLC*), but the importance of this as a trigger for vernalization is still in debate [32], [34]. Using tiling arrays, it was found that 24% of regulated protein coding genes were controlled by the circadian clock, while 7% of the protein coding genes have circadian-regulated NATs in Arabidopsis [35].

Here we show that commercial sugarcane varieties have robust circadian rhythms driven by a central oscillator that is similar, but not identical to the Arabidopsis circadian clock. We also show that the proportion of probes that had rhythmic time courses s higher than the ones found in other plants and that the transcript levels in both sense and antisense directions are regulated by the circadian clock. We also show that a high proportion of probes associated with the harvesting and storage of energy from light and probes associated with DNA, RNA and protein synthesis are regulated by the clock circadian but the former are expressed during the light phase of the day and the latter are expressed during the dark phase. Taken together, our data suggest that the circadian clock is highly active in commercial sugarcane varieties and may be important to its high productivity and sucrose accumulation.

## Results

### A significant proportion of the sugarcane transcriptome is circadian-regulated

Three month old sugarcane plants (RB855453) were entrained in a 12 h light/12 h dark photoperiod and transferred to continuous light for 24 h before being harvested every 4 h for 48 h. RNA extracted from the harvested samples was hybridized in 44 k custom oligoarrays [29]. Of the 14,119 probes with positive signals for transcripts in the sense direction (SS), 75.7% were above the noise levels and were considered expressed (10,691±541; mean ± SD; n = 12). Of the 6,575 probes for transcripts in the antisense direction (AS) with positive signal, 19.7% were considered expressed (1,297±450; n = 12). Only time courses of probes considered expressed in at least 10 of the 12 arrays were analysed for the presence of circadian behaviour. The dataset of expressed probes corresponded to 9,931 SS (70.3%) and 665 AS (10.1%) (Table 1), a similar number to what was found in previous experiments [29].

**Table 1:** Number of rhythmic probes in the sense direction (SS) and in the antisense direction (AS) considered expressed and rhythmic in the sugarcane dataset.

	SS	SS (%)	AS	AS (%)
**Number of probes**	14,119	-	6,575	-
**Probes expressed in ≤1 time point**	11,893	84.2*	2,352	35.4*
**Probes expressed in ≤10 time points**	9,931	70.3*	665	10.1*
**Rhythmic probes**	3,189	32.1**	146	21.8**

* compared to the number of probes;

** compared to number of probes expressed in ≤10 time points.

**Figure 1:**
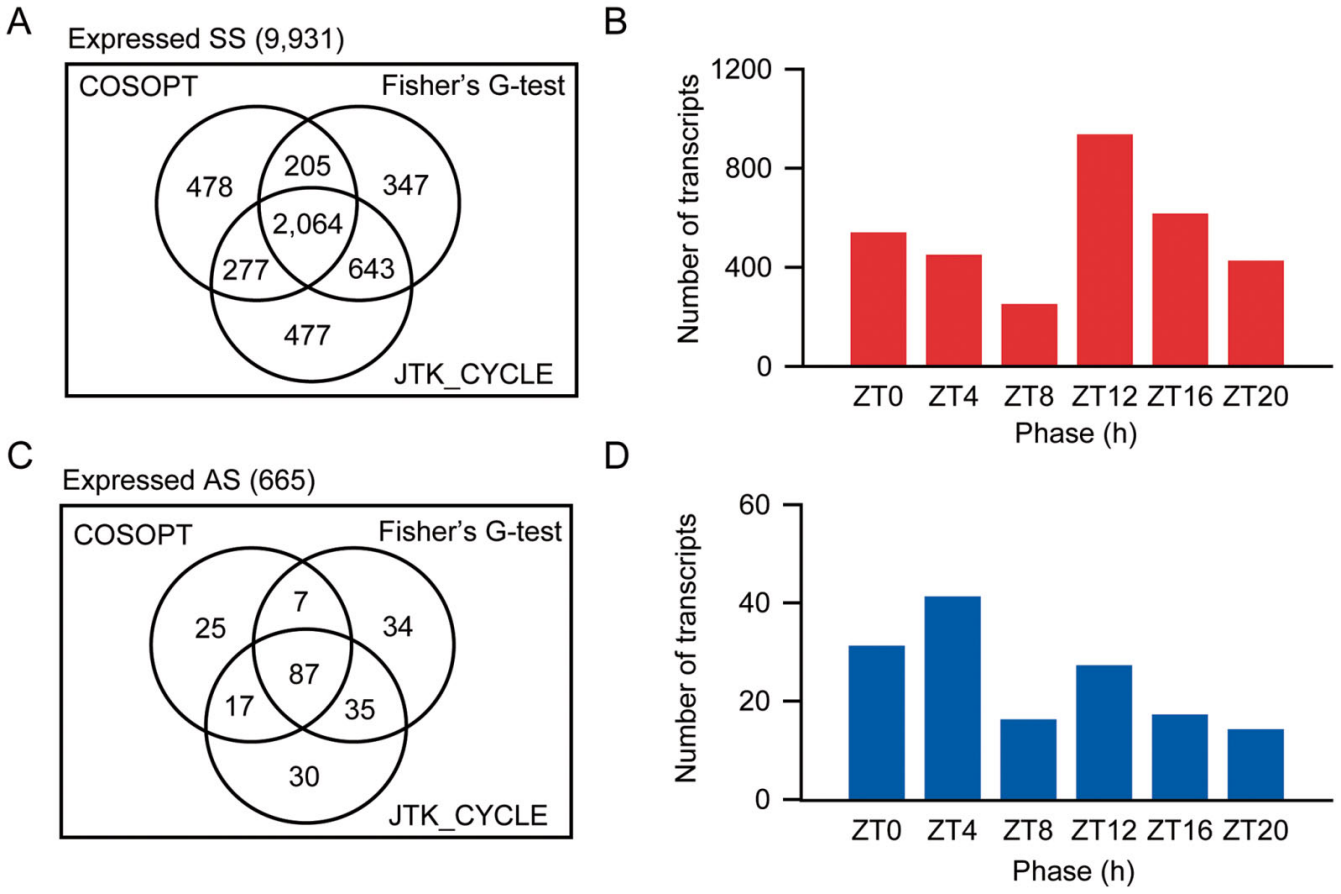
Identification of rhythmic sense and antisense transcripts in sugarcane. (**A**) Venn diagram showing the number of sense probes that were considered expressed and were considered rhythmic by three algorithms: JTK_CYCLE, COSOPT and Fisher's G-test. Probes identified as rhythmic in at least two algorithms were considered circadian. (**B**) Phase distribution of rhythmic sense probes. The phase of each rhythmic probe was estimated using JTK_CYCLE and binned in 6 groups, according to the time of their peak. (**C**) Venn diagram showing the number of antisense probes that were considered expressed and considered rhythmic by three algorithms: JTK_CYCLE, COSOPT and Fisher's G-test. (**D**) Phase distribution of circadian antisense probes.

Three different methods were used to identify time courses that showed rhythms with a circadian period (20 h to 28 h): COSOPT, JTK_CYCLE and Fisher's G-test (Figure 1) [36]–[38]. COSOPT considered rhythmic (p<0.05) a total of 3,024 SS time courses (30.4%) and a total of 136 AS time courses (20.5%). Fisher's G-test considered rhythmic (p<0.05) a total of 3,259 SS time courses (32.8%) and a total of 163 AS time courses (24.5%). Finally, JTK_CYCLE considered rhythmic 3,461 SS (34.9%) and 169 AS (25.4%) time courses. As each algorithm has its own biases, in order to reduce the number of false positives and false negatives, only the time courses that were considered as having a circadian rhythm by more than one method were counted. In total, 3,189 SS (32.1%) and 146 AS (22.0%) were considered circadian clock regulated. In order to compare these results with previous studies that used similar experimental conditions, we have reanalysed one maize, one rice and two Arabidopsis datasets [17], [18], [20], [39]. The maize dataset had 4.1% of their probes considered circadian clock regulated, the rice dataset had 2.1%, the Arabidopsis (Covington; C) dataset had 10.7%, and the Arabidopsis (Edwards; E) dataset had 12.4% (Figure S1; Table 2).

**Table 2:** Proportion of rhythmic probes in different plant species using different algorithms.

	circadian probes in original paper	method of analysis	rhythmic probes using our method	rhythmic ortholog clusters in array
**Sugarcane**	-	-	3,189 (32.1%)	2,027 (36.9%)
**Maize** 1	1,444 (10.8%)	HAYSTACK + COSOPT	717 (4.1%)	58 (11.7%)
**Rice** 2	7,230^5^ (12.6%)	HAYSTACK	1,198 (2.1%)	508 (7.8%)
**Arabidopsis (C)** 3	1,610 (10.4%)	MBEI	2,445 (10.7%)	1,473 (18.2%)
**Arabidopsis (E)** 4	3,503 (15.4%)	COSOPT	2,822 (12.4%)	1,775 (21.9%)

1 Khan et al. [18];

2 Filichkin et al. [17];

3 Covington and Harmer [39];

4 Edwards et al. [20];

5 estimated.

COSOPT was used to estimate the period of circadian time courses. The circadian SS time courses had a mean period of 25.0±2.50 h (mean ± SD; n = 3161) and the circadian AS time courses had a mean period of 24.6±2.63 h (n = 144). The phases of the circadian time courses were estimated using JTK_CYCLE. The results were divided into six groups according to their estimated time of peak. Circadian time courses were assigned to all phases. Most of the circadian SS time courses (29.2%) had a peak 12 h after the subjective dawn (*zeitgeber* time 12; ZT12), while most of the circadian AS time courses (28.1%) had a peak at early morning (ZT4) (Figure 1B and D).

### More ortholog clusters are circadian-regulated in sugarcane than in other plants

Our oligoarray was designed based on the sugarcane expressed sequence tag (SUCEST) database. The SUCEST database has 43,141 putative transcripts known as sugarcane assembled sequences (SAS) [40]. Every probe that passed quality controls and was unique to one SAS was used, minimizing any selection bias. However, there are several SAS within SUCEST that may correspond to the same coding gene, either because they represent different alleles, different paralogs, or because they are fragments of the same gene that were not assembled together (Figure S2). Furthermore, each probe was not designed to differentiate among alleles or duplications of the same gene. Thus, it was possible that the large proportions of circadian-regulated probes that we had identified did not correspond to a higher proportion of circadian-regulated transcripts. In order to address this issue, we have selected 9 enzyme models associated with sucrose metabolism and compared their time courses among the different datasets. Eight of the sugarcane enzyme models had at least one rhythmic probe. In contrast, four to six Arabidopsis enzyme models and one to three rice and maize enzyme models had at least had at least one rhythmic probe (Table 3).

**Table 3:** Sugarcane has a high proportion of rhythmic probes associated with sucrose metabolism.

	Sc	Arabidopsis			Rice		Maize	
dataset/method of analysis	CJF	C-CJF	E-CJF	D	CJF	D	CJF	D
sucrose-phosphate synthase	3 (3)^1^	1 (3)^4^	1 (3)^4^	1 (3)^4^	0 (3)	0 (3)	0 (3)	0 (3)
sucrose phosphatase	1 (2)^1^	1 (2)^1^	1 (2)^4^	1 (2)^4^	0 (2)	1 (2)^3^	0 (2)	0 (2)
UDPG pyrophosphorylase	1 (1)^1^	1 (2)^1^	2 (2)^4^	1 (2)^4^	0 (3)	0 (3)	0 (1)	1 (1)^1^
fructokinase	2 (2)^1^	0 (7)	0 (7)	0 (7)	0 (2)	0 (2)	1 (2)^1^	0 (2)
sucrose synthase	0 (2)	0 (6)	0 (6)	0 (6)	0 (4)	0 (4)	0 (3)	0 (3)
neutral invertase	1 (2)^2^	1 (3)^1^	0 (3)	1 (3)^2^	0 (2)	1 (2)^3^	0 (0)	0 (0)
hexokinase	1 (1)^2^	0 (7)	0 (7)	3 (7)	0 (2)	0 (2)	1 (3)^1^	0 (3)
glucose-6-phosphate isomerase	1 (1)^2^	0 (2)	0 (2)	0 (2)	2 (3)^1^	1 (3)^1^	0 (0)	0 (0)
phosphoglucomutase	1 (2)^3^	1 (3)^3^	2 (3)^3^	2 (3)^3^	0 (2)	0 (2)	0 (3)	0 (3)
**Total**	11 (16)	5 (35)	6 (35)	9 (35)	2 (23)	3 (23)	2 (17)	1 (17)

The number of circadian probes and the total number of probes (in parenthesis) was determined for each enzyme model in different datasets and different methods of analysis. CJF: analysis using COSPOT, Fisher's G-test and JTK-CYCLE; C-CJF: Covington dataset; E-CJF: Edwards dataset; D: data taken from DIURNAL (http://diurnal.mocklerlab.org/) [76].

1 probes that peak at ZT0;

2 cells in red represent probes that peak at ZT8;

3 probes that peak at ZT16;

4 probes that peak at ZT20.

We also have clustered together orthologs and inparalogs of the species we have circadian datasets and only compared those that were common among then. An ortholog catalogue of 18,611 ortholog clusters was created using a combination of InParanoid with MultiParanoid [41], [42]. InParanoid was used to identify orthologs and inparalogs in pairwise proteome comparisons of five different species: 39,021 proteins from sugarcane, 36,338 proteins from sorghum, 106,046 proteins from maize, 51,258 proteins from rice, and 35,386 proteins from Arabidopsis [41]. MultiParanoid was used to merge InParanoid pairwise comparisons into multi-species ortholog clusters [41]. Thus, an ortholog cluster is made of all the orthologs and inparalogs identified among the plant species used. Inparalogs are genes in which duplication happened after the speciation event, in contrast to outparalogs. A caveat of this analysis is that all the paralogs generated from duplication events that occurred after the split from Arabidopsis and the grasses common ancestor were considered inparalogs [42]. However, the incorporation of these paralogs to ortholog clusters should minimize the problem with inaccurate or incomplete sequences assemblies, a necessity when using data from a species whose genome that has not been completely sequenced like the sugarcane [41].

We have validated the ortholog clusters by comparing the manual annotation of 9 genes associated with sucrose metabolism (Table S1). Of the 178 annotated genes that were grouped in 28 ortholog clusters, only 2 were false positives and did not correspond to the annotated enzyme model. There were 31 incomplete SAS that were not assigned ortholog clusters but could be annotated manually. Among the other species, there were 17 genes that were false negatives and should be included in an ortholog cluster but were not included in any.

**Figure 2:**
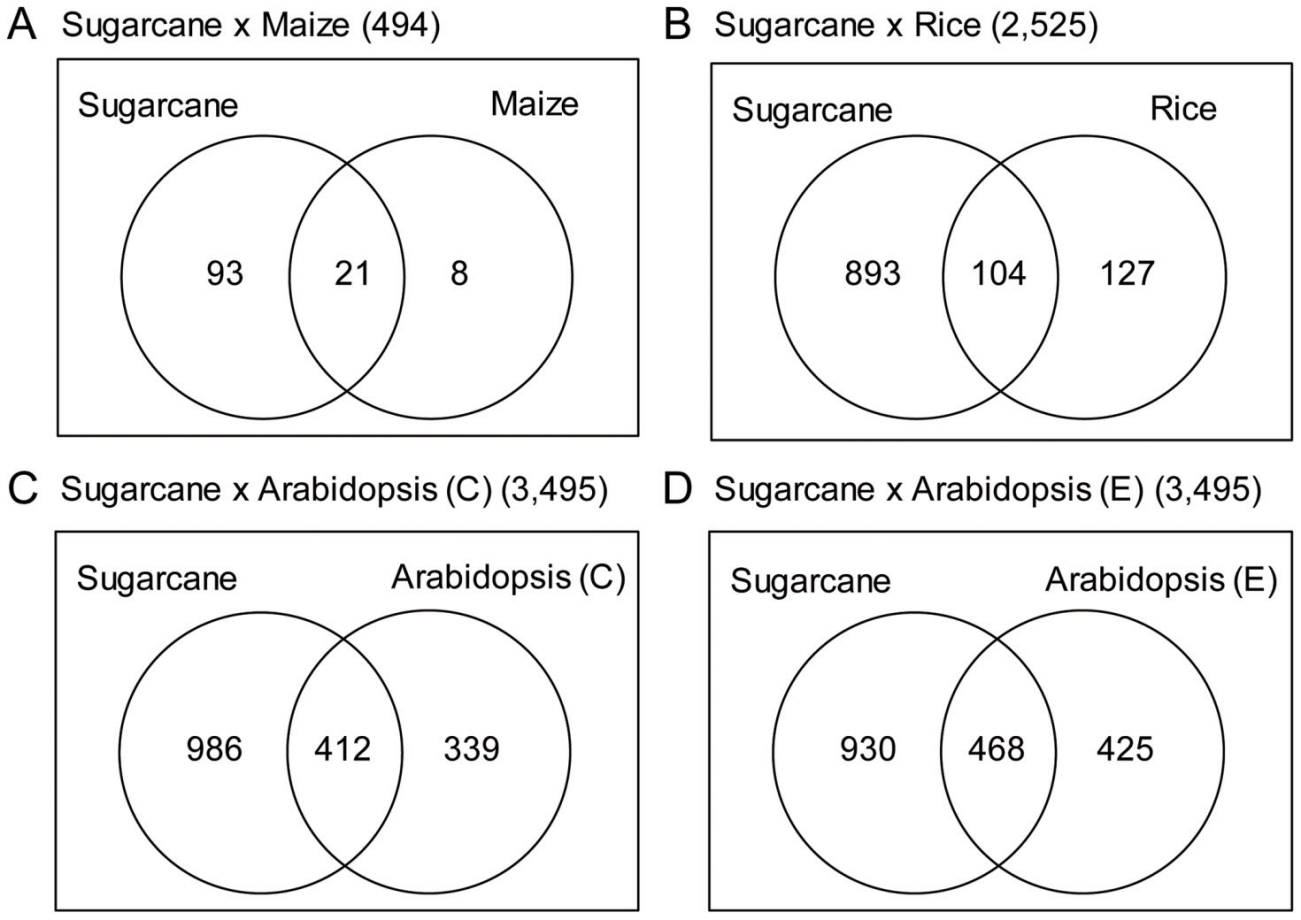
The sugarcane dataset has a higher proportion of circadian ortholog clusters in pairwise comparisons with other dataset. A list of common ortholog clusters was made for each pairwise comparison between the sugarcane dataset and (**A**) the maize dataset, (**B**) the rice dataset, (**C**) the Arabidopsis (Covington) dataset and (**D**) the Arabidopsis (Edwards) dataset. A Venn diagram was made for each comparison to show overlap between circadian ortholog clusters of each dataset used in the comparison.

The ortholog catalogue allowed us to associate the probes of each array to an ortholog cluster. This way, we can use the probes for ortholog clusters that are common to a pair of arrays, eliminating any selection biases that exist in each array. For example, there were 246 ortholog clusters present in both sugarcane dataset and maize dataset. Of these, 114 (46.3%) were considered circadian in sugarcane dataset and 29 (11.8%) were considered circadian in the maize dataset (Figure 2). In total, the sugarcane array had 5,490 ortholog clusters, 2,027 of which (36.9%) were considered circadian, a greater proportion than the other plant datasets (Table 2).

We also compared if the phase ortholog clusters was conserved in different species. A pairwise comparison using circadian orthologs groups that were common to a pair of arrays showed that the highest coefficient of determination (r^2^) was between the two Arabidopsis datasets (r^2^ = 0.72). Among the comparisons that used the sugarcane database, the highest r^2^ was between sugarcane and maize (r^2^ = 0.67) (Table 4).

**Table 4:** Coefficient of determination (r^2^) between the phases of ortholog clusters considered rhythmic in two arrays.

	Maize	Rice	Arabidopsis (E)	Arabidopsis (C)
**Sugarcane**	0.67 (21)	0.34 (104)	0.34 (468)	0.36 (412)
**Arabidopsis (C)**	0.54 (14)	0.29 (101)	0.72 (881)	
**Arabidopsis (E)**	0.40 (17)	0.31 (114)		
**Rice**	0.62 (5)			

In parenthesis is the number of ortholog clusters pairs used in each comparison.

### Antisense transcripts are controlled by the circadian clock

**Figure 3:**
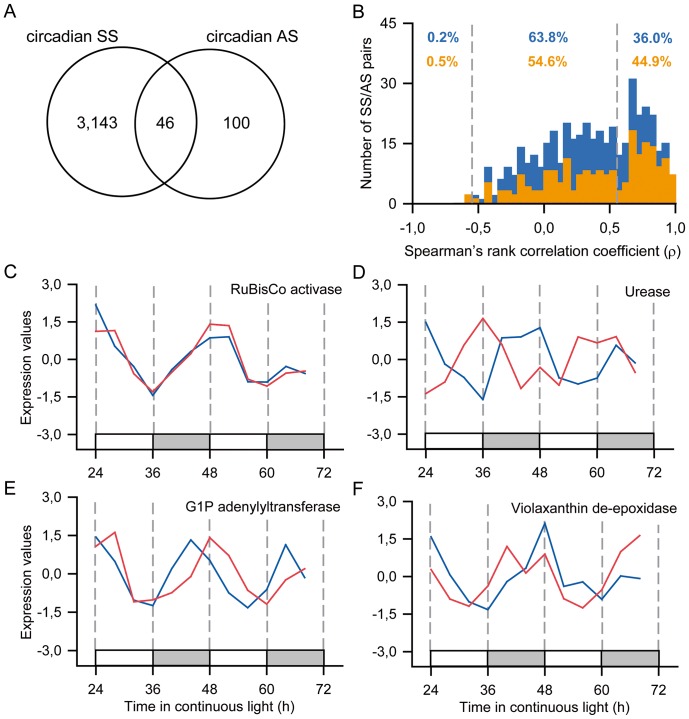
Sense and antisense transcripts are modulated differently by the circadian clock. (**A**) Overlap between SAS that had their probes for sense transcripts (SS) considered circadian and SAS that had their probes for antisense transcripts (AS) considered circadian. (**B**) Distribution of Spearman's rank correlation coefficient (ρ) for each of all the 428 pairs of SS/AS (light blue) and only the 207 pairs of SS/AS that had at least one probe considered circadian (orange). If ρ >0.56, correlation is positive and significant. If ρ <−0.56, correlation is negative and significant. (**C to F**) Z-score normalized expression levels of SS (red) and AS (dark blue) for a gene that have (**C**) both SS and AS in the same phase (RuBisCo activase; SCBGLR1044D06.g,), (**D**) SS and AS in opposite phases (urease, SCSGLR1045A02.g), (**E**) AS peaking before SS (glucose 1-phosphate adenylyltransferase, SCVPCL6061A06.g) and (**F**) SS peaking before AS (violaxanthin de-epoxidase, SCVPHR1095C07.g). White boxes represent periods of subjective day and light grey boxes represent periods of subjective night.

There was little overlap between the 3,189 SS and 146 AS transcripts identified as circadian clock regulated (Figure 3A). This would suggest little correlation between SS and AS expression. However, when the Spearman's rank correlation coefficient (ρ) was calculated for each of the 428 SS/AS transcript pairs, 36% of the pairs had a positive correlation (Figure 3B). If only the 207 SS/AS pairs that had at least one transcript considered circadian clock regulated were considered, 44.9% of the pairs had a positive correlation. The ρ coefficient is a non-parametric measure of the dependence between two variables. Values of ρ above 0.56 indicate a significant positive correlation between the SS and the AS. Values bellow −0.56 indicate a significant negative correlation. The distribution of Spearman's rank correlation coefficient among AS/SS pairs in the oligoarray was bimodal (Figure 3B). This suggested that there were two types of control of AS expression: one that regulated both SS and AS expression and one that regulated AS expression independently of SS expression. RuBisCo activase (SCBGLR1044D06.g) was an example of SAS with SS and AS time courses very similar to each other (Figure 3C). On the other hand, a urease (SCSGLR1045A02.g) had its SS and AS time courses with completely opposite phases (Figure 3D). A glucose 1-phosphate adenylyltransferase (SCVPCL6061A06.g) and a violaxanthin de-epoxidase (SCVPHR1095C07.g) were examples of SAS that had their AS and SS time courses with phases a few hours apart (Figure 3E and F).

### Components of circadian clock have strong rhythms

**Figure 4:**
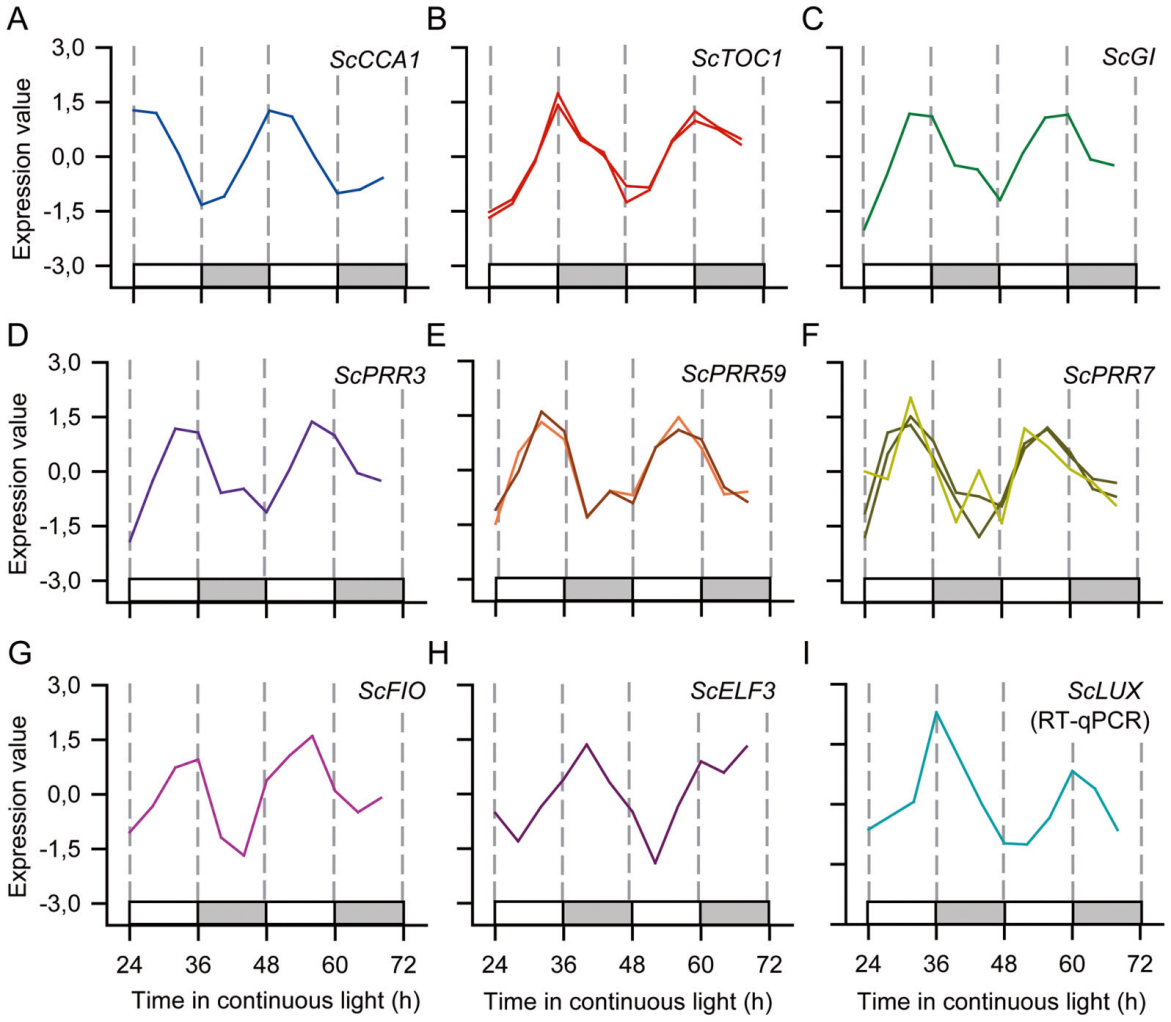
Time courses of probes associated with the central oscillator. Z-score normalized time courses of rhythmic probes for sense sugarcane transcripts associated with the central oscillator. (**A–H**) Expression levels were measured using oligoarrays. Different probes for the same sugarcane genes are represented separately. Lighter lines indicate probes for antisense transcripts expression of the gene. (**I**) Expression levels were measured using RT-qPCR, using an arrhythmic GAPDH as reference. (**A**) *CIRCADIAN ASSOCIATED1* (*ScCCA1*), (**B**) *TIME OF CHLOROPHYLL A/B BINDING PROTEIN EXPRESSION (ScTOC1*), (**C**) *GIGANTEA* (*ScGI*), (**D**) *PSEUDO-RESPONSE REGULATOR3* (*ScPRR3*), (**E**) *ScPRR59*, (**F**) *Sc*PRR7, (**G**) *FIONA (ScFIO)*, (**H**) *EARLY-FLOWERING3* (Sc*ELF3*), and (**I**) *LUX ARRHYTHMO* (*ScLUX*). White boxes represent periods of subjective day and light grey boxes represent periods of subjective night.

Arabidopsis circadian clock genes were used to identify putative orthologs in SUCEST. Rice circadian clock genes were used to confirm the identified orthologs. There was only one sequence found to be similar to both *AtCCA1* and *AtLHY*. *ScLHY* (*ScMYB19*; SCCCLR1048E10.g) had strong rhythms that peaked at ZT1 (Figure 4A). Validation using qPCR showed that the *ScLHY* expression levels at its peak were 44.8 to 63.4 times higher than expression levels at its trough (Figure S3). Two *ScTOC1* probes (SCCCSB1002H04.g and SCEPLB1042B08.g) were present in the array, peaking at ZT13 and ZT14 respectively (Figure 4B). These probes were designed from two different SAS that match different portions of one gene (Figure S2). *ScGI* (SCJFAD1014B07.b) peaked at ZT12, while *ScPRR3* (SCACLR1057G02.g), *ScPRR59* (SCCCLR1077F09.g) and *ScPRR7* (SCACLR1057C07.g and SCCCST3001B11.g) peaked at ZT10 (Figure 4C to F). *ScPRR7* and *ScPRR59* expression in the AS direction was detected and they correlated well with the expression in the SS direction (Figure 4E and F). Sugarcane candidates for *AtFIO1* (SCBGLR1096A01.g), *AtELF3* (SCEZLB1009F09.g), and *AtLUX* (SCMCST1052A09.g) [43], [44] were also identified and showed circadian expression, with peaks at ZT10, ZT16 and ZT12, respectively (Figure 4G to I). No sugarcane candidates for *AtELF4*and *AtCHE* [4], [6], [45] were identified in the SUCEST database or in our unpublished draft of the sugarcane genome. In sugarcane, as in other monocots, the sequences with highest identity to *AtELF4* are more similar to Arabidopsis *ELF4-like* (*AtEFL*) than *AtELF4* [46], [47]. Likewise, the sequences with highest identity to *AtCHE* are more similarity to other Arabidopsis TCP-family transcription factors than *AtCHE*.

**Figure 5:**
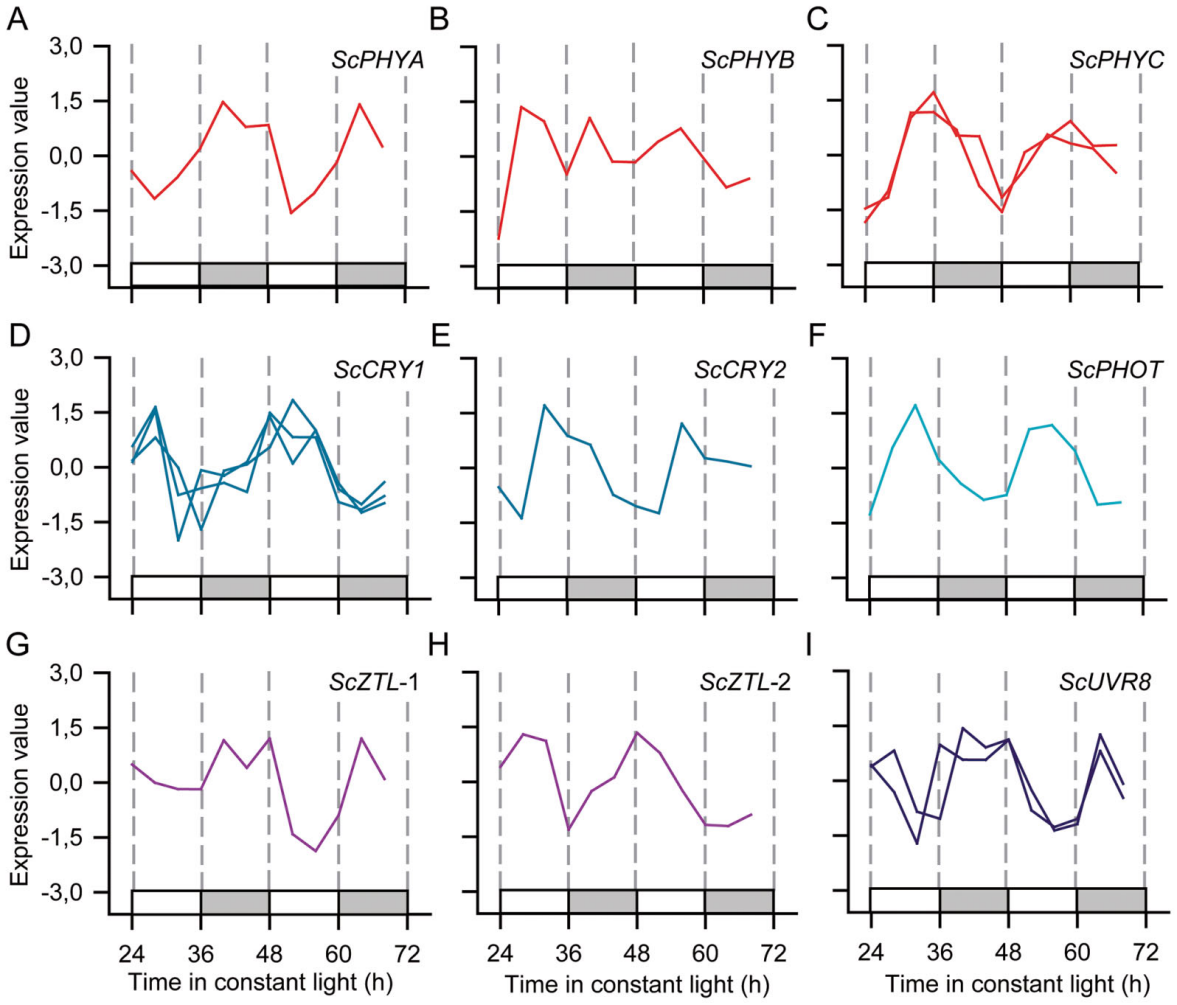
Time courses of probes associated with the perception of light. Z-score normalized time courses of rhythmic probes for sugarcane transcripts associated with the perception of light. Expression levels were measured using oligoarrays. Different probes for the same sugarcane genes are represented separately. (**A**) *PHYTOCHROME A* (Sc*PHYA*), (**B**) *ScPHYB*, (**C**) Sc*PHYC*, (**D**) *CRYPTOCHROME1* (Sc*CRY1*), (**E**) Sc*CRY2*, (**F**) *PHOTOTROPIN* (*ScPHOT*) (**G**) *ZEITLUPE* (*ZTL-1*), (**H**) *ZTL*-2, (**I**) Sc*UVR8*. White boxes represent periods of subjective day and light grey boxes represent periods of subjective night.

We also have identified putative sugarcane photoreceptors: four red-light receptors *PHYTOCHROME*, *ScPHYA* (SCCCCL3080H06.g), *ScPHYB* (SCQSLR1040D12.g), *ScPHYC*-1 (SCCCLR1065C10.g) and *ScPHYC*-2 (SCVPRT2081G11.g); two *CRYPTOCHROME*, *ScCRY1* (SCAGST3138B05.g, SCCCRZ1C02F07.g and SCBFRZ3009A01.g) and *ScCRY2* (SCRFST1042F05.g and SCSBAD1050G03.g); two *ZEITLUPE*, *ScZTL*-1 (SCCCLR1C07F05.g) and *ScZTL*-2 (SCCCLB1025H12.g); one *PHOTOTROPIN*, *ScPHOT* (SCCCRZ3001D06.g); and one ultraviolet-B receptor *ScUVR8* (SCSBAD1054F04.g and SCBGFL4056D06.g) [48], [49]. The expression of all putative sugarcane photoreceptors but *ScPHYB* and *UVR8* were considered circadian (Figure 5). *ScPHYA* had a peak at ZT17 while both *ScPHYC* had a peak at ZT12 (Figure 5A to C). The three *ScCRY1* probes for the same gene showed closely related profiles and all peaked between ZT22 and ZT0, showing that the arrays show technical reproducibility (Figure 5C). *ScCRY2* had a peak at ZT17 (Figure 5D). The two *ScZTL* peaked at ZT20 and ZT2 while the *ScPHOT* peaked at ZT8 (Figure 5F to H).

### Outputs of the circadian clock cluster by function

We have identified important biological processes in plants that have a high proportion of rhythmic probes and compared their phases. Some biological processes had a noticeable number of rhythmic probes that clustered around the same phase. For example: photosynthesis, carbohydrate metabolism, hormone signalling and genetic information processing (Table 5). Other important processes that had many rhythmic probes were: lipid metabolism, amino acid metabolism, nitrogen metabolism and flowering regulation (Figure S4). Indeed, a term enrichment analysis of the annotation of the rhythmic probes had ‘Protein Metabolism’, ‘Others’ and ‘RNA metabolism’, as enriched function categories among the circadian SS (Table S2) and ‘Porphyrin and chlorophyll metabolism’, ‘Protein metabolism’ and ‘Cytoskeleton and vesicle transport' (Table S3) as enriched function categories among the circadian AS, if the ‘Unknown’ and ‘No match’ categories are not considered.

**Table 5:** Pathways regulated by the sugarcane circadian clock.

	Rhythmic SS	Rhythmic AS	Phase (h)
**Photosynthesis**			
Light harvesting complex	8 (8)	0 (1)	6±1.5
Photosystem I	3 (7)	0 (3)	4±0.0
Photosystem II	5 (11)	0 (2)	0±0.0
Photosynthetic electron transport	2 (13)	2 (3)	22±0.5
Chlorophyll synthesis	4 (5)	2 (2)	3±2.0
Carotenoid and xanthophyll synthesis	8 (9)	0 (2)	0±0.0
C4 carbon accumulation	12 (32)	2 (3)	0±2.0
Calvin cycle	9 (22)	0 (6)	0±2.0
Photorespiration	11 (21)	0 (5)	1±3.0
**Carbohydrate metabolism**			
Sucrose synthesis	7 (9)	0 (2)	2±1.0
Sucrose degradation	2 (9)	1 (1)	8±0.0
Starch synthesis	6 (9)	3 (3)	3±1.0
Starch branching	2 (3)	0 (0)	11±2.0
Starch degradation	5 (6)	1 (0)	15±3.0
Starch debranching	4 (4)	0 (0)	21±1.0
Glycolysis/Gluconeogenesis	5/11 (32)*	0 (1)	18±5.0/22±3.5*
Citric acid cycle	7 (31)	0 (3)	17±4.0
Lipid metabolism	9/7 (39)	0 (1)	0±2.0/14±2.0*
**Amino acid metabolism**	37/18 (91)	1 (8)	0±2.0/14±2.0*
**Hormone signalling**			
Auxin synthesis	4 (11)	1 (1)	16±1.0
Auxin response	5/6 (28)*	1 (1)	9±1.0/16±0.0*
ABA synthesis	1/2 (6)*	1 (1)	14±0.0/22±2.0*
ABA response	12/3 (30)*	2 (2)	1.5±1.5/12±0.0*
**Genetic Information Processing**			
DNA replication	10 (19)	0 (0)	14±2.0
Histones and histone regulation	23 (67)	4 (41)	14±2.0
RNA polymerase	14 (31)	0 (3)	13±1.0
Spliceosome	19 (84)	0 (4)	12±2.0
RNA degradation	14 (42)	0 (2)	14±2.0
RNA transport	19 (69)	0 (1)	12±2.0
Ribosome biogenesis	27 (51)	0 (1)	12±0.7
Protein synthesis	119 (206)	11 (12)	14±2.0
Protein degradation	12 (83)	3 (4)	16±4.0
Protein transport	9 (29)	0 (1)	18±4.0

The number of SS and AS probes considered circadian compared to the total number of transcripts expressed in the pathway (in parenthesis) and the phase of the circadian SS transcripts (median ± MAD).

* two populations with distinct phases were detected.

**Figure 6:**
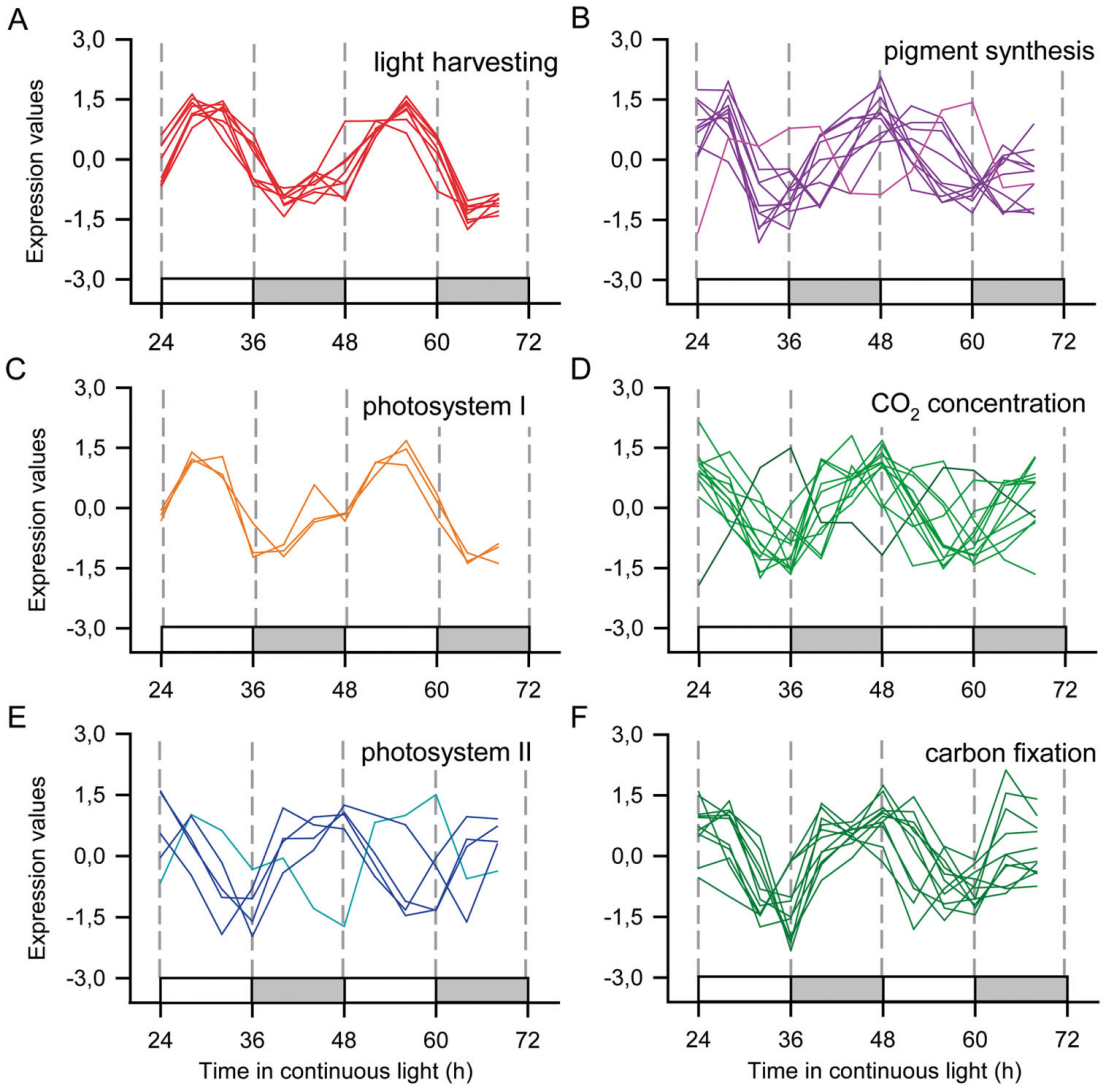
Rhythmic probes associated with the photosynthetic pathways. Z-score normalized time courses of rhythmic probes for transcripts associated with the photosynthetic pathway were separated into (**A**) light harvesting complex (red); (**B**) chlorophyll, carotenoid and xanthophyll synthesis (purple); (**C**) photosystem I (orange); (**D**) CO_2_ concentration mechanisms (light); (**E**) photosystem II (blue); and (**F**) carbon fixation mechanisms (dark green). Lines in different colours indicate probes with contrasting phases. White boxes represent periods of subjective day and light grey boxes represent periods of subjective night.

One important feature of the outputs of the circadian clock is that transcripts that are associated with the same biological process are co-regulated. For example, probes associated with light-dependent reactions of photosynthesis had a tendency to peak between ZT0 and ZT6, while probes associated with the carbon accumulation and fixation genes had a tendency to peak between ZT20 and ZT02 (Figure 6). We have used median and median absolute deviation (MAD) in order to describe the phase of a population of probes associated with the same biological process. These parameters are more resistant to outliers than mean and standard deviation. For example, an outlier probe that has a phase of ZT18 may have a great impact in the average of a group of probes with phase of ZT0. MAD is a measure of the population variability, it is defined as the median of the difference between each value and the median of whole the population. This way, half of the values in the population are within one MAD of the median.

All of the eight probes involved in the light harvesting complex were considered rhythmic and peaked at ZT6±1.5 (median ± MAD; n = 8; Figure 6A). Three of the 7 probes of the photosystem I peaked at ZT4±0.0 (n = 3; Figure 6C), while five of the 11 probes of the photosystem II peaked at ZT0±0.0 (n = 5), with one outlier peaking at ZT10 (Figure 6E). Moreover, four of five probes associated with chlorophyll synthesis peaked at ZT3±2.0 and 8 of 9 (n = 4) probes associated with carotenoid and xanthophyll synthesis peaked at ZT0±0.0 (n = 8; Figure 6B). Twelve probes associated with the CO_2_ accumulation processes of C4 plants were considered circadian and peaked at ZT0±2.0 (with two outliers, at ZT12 and at ZT18; n = 12) (Figure 6D), and 9 probes associated with the Calvin cycle at ZT0±2.0 (n = 9; Figure 6F).

**Figure 7:**
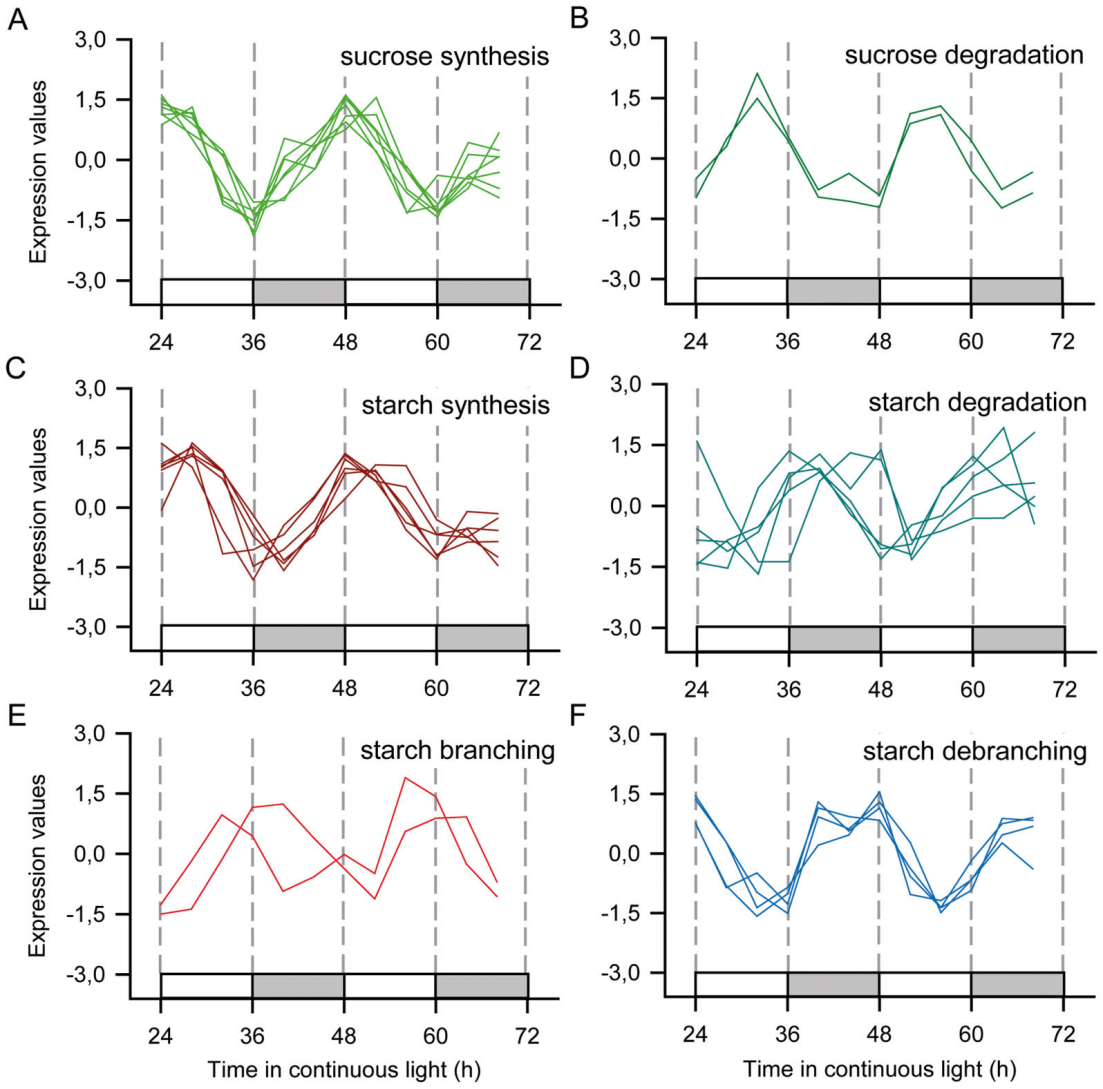
Rhythmic probes associated with sugar storage. Z-score normalized time courses of rhythmic probes for transcripts associated with sugar storage pathways were separated into (**A**) sucrose synthesis (light green); (**B**) sucrose degradation (dark green); (**C**) starch synthesis (dark red); (**D**) starch degradation (cyan); (**E**) starch branching (red); and starch debranching (light blue). White boxes represent periods of subjective day and light grey boxes represent periods of subjective night.

Another important feature of circadian clocks is that the phase of some transcripts is similar to the phase of the different physiological processes that happen during the course of a day. For example, sugar metabolism has probes associated with sucrose and starch synthesis peaking in the subjective morning (ZT2±1.0, n = 7; and ZT3±1.0, n = 6, respectively); sucrose degradation during the subjective evening (ZT8±0.0; n = 2); and starch degradation during the early subjective night (ZT15±3.0; n = 5) (Figure 7A to D). Curiously, starch branching probes also peaked during the early subjective night (ZT11±2.0; n = 2) while starch debranching probes peaked during the late subjective night (ZT21±1.0; n = 4) (Figure 7E and F). Photosynthesis and sucrose metabolism are linked together by the upper part of the glycolysis/gluconeogenesis pathway (ZT22±3.5; n = 11), that has a phase that matches the phases of the carbon fixation and sucrose synthesis pathways. On the other hand, probes associated with the lower part of the glycolysis/gluconeogenesis pathway have a phase (ZT18±5.0; n = 5) that matches the transcripts for citric acid cycle (ZT17±4.0; n = 7).

During the subjective night, there were a significant number of probes that were associated with the processing of genetic information, while there were a significant number of probes associated with photosynthesis and the use of fixed carbon during the subjective day (Table 5). Probes associated with histones and their regulatory proteins peak at ZT14±2.0 (n = 23), probes associated with ribosome biogenesis peak very tightly at ZT12±0.7 (n = 27), and a very large number of probes associated with the synthesis of new proteins peak at ZT14±2.0 (n = 119).

**Figure 8:**
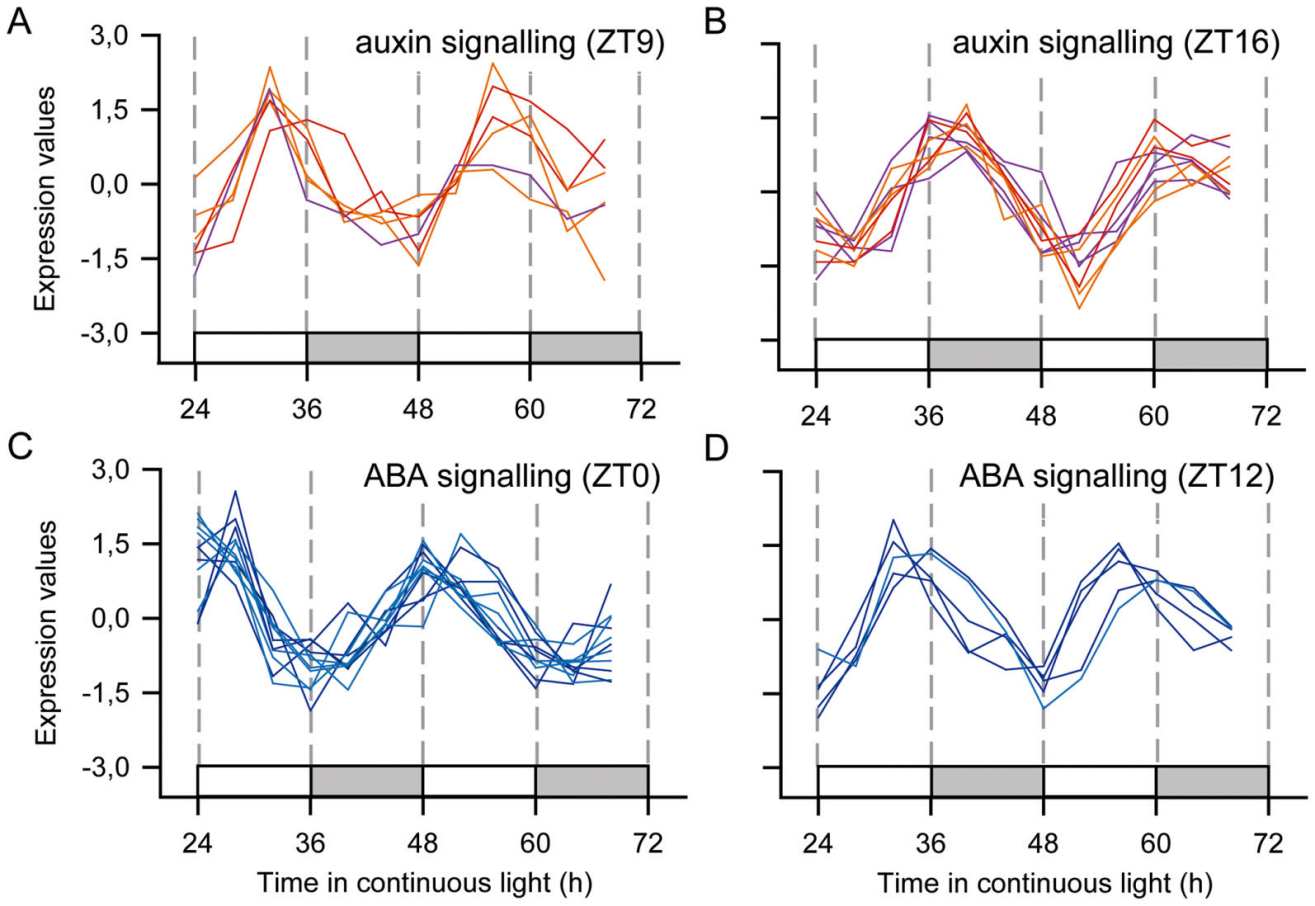
Rhythmic probes associated with auxin and ABA signalling. Z-score normalized time courses of rhythmic probes for transcripts associated with auxin and ABA signalling were separated into their approximated phase: (**A**) transcripts associated with auxin signalling peaking close to ZT9; (**B**) transcripts associated with auxin signalling peaking close to ZT16; (**C**) transcripts associated with ABA signalling peaking close to ZT0; and (**D**) transcripts associated with ABA signalling peaking close to ZT12. (**A–B**) Transcripts associated with auxin synthesis and reception (purple), ARF (orange), and Aux/IAA (red). (**C–D**) Transcripts associated with ABA synthesis and reception (dark blue) and transcripts associated with ABA response (blue). White boxes represent periods of subjective day and light grey boxes represent periods of subjective night.

We also found evidence of control of hormone signalling by the circadian clock (Figure 8A and B). Five auxin synthesis and transport probes peaked between ZT14 and ZT18. Accordingly, four AUX/IAA transcripts and two ARF probes that are part of the auxin response pathways peak between ZT14 and ZT17. However, there were two ARF probes, two AUX/IAA probes and one auxin receptor probe that peaked between ZT7 andZT10, suggesting that there were two times during the day when auxin signalling was at a peak. Probes associated with ABA signalling were also regulated by the circadian clock in more than one phase (Figure 8C and D). One probe involved in ABA synthesis had a peak in the subjective early morning (ZT2), while two had a peak during the subjective night (ZT14 and ZT17). On the other hand, probes associated with ABA response peak close to subjective dawn (ZT20 to ZT4) and close to subjective dusk (ZT10 to ZT12). Ethylene and brassinosteroid signalling also had rhythmic probes that may suggest that the whole pathway may be controlled by the circadian clock (Figure S5). We found no evidence of significant control of the circadian clock over cytokinin and gibberellin signalling pathways.

## Discussion

Commercial sugarcane is the product of the hybridization of two polyploid species, *Saccharum officinarum* and *S. spontaneum*, that resulted in 8 to 10 copies of each gene in its genome [22], [50]. However, the sugarcane circadian clock is still capable of generating periods close to 24 h. There is evidence of sugarcane circadian clock control of over 32.1% of the probes for expressed sense transcripts (SS) and 22.0% of the probes for expressed antisense transcripts (AS) in plants grown under light cycles and constant temperature (Table 1). In this study, we were unable to design probes that could distinguish among paralogs, mainly because we lack the genomic resources. This means that each rhythmic probe has at least one transcript that is controlled by the circadian clock. At least three scenarios could happen that may bias the proportion of rhythmic probes: i) several different genes may hybridize the same probe at the same time, which could mask rhythms of paralogs that have very different phases; ii) only one rhythmic transcript among several others that hybridize to the same probe may be enough to make the probe to be marked as rhythmic; and iii) a rhythmic transcript may hybridize to more than one probe. These issues will only be completely clarified whenever the sugarcane genome, with all its copies, is sequenced and the transcription levels of different copies of the same gene can be measured.

We have tested whether bias could explain the large proportion of rhythmic probes in our dataset. A selection of nine enzyme models associated with sucrose metabolism showed that sugarcane had more enzyme models with at least one rhythmic probe than the other datasets. We also grouped probes into ortholog groups that were common among the compared datasets and sugarcane had more rhythmic ortholog clusters than the compared datasets (Table 2). Thus, it is possible that the sugarcane circadian clock controls a high proportion of the sugarcane transcriptome. This could be the result of the multiple intra and interspecific hybridizations and intensive selection for desired agronomic traits that increased ploidy and aneuploidy levels in this plant. In particular, the hybridization between *S. officinarum* and *S. spontaneum* could result in the enrichment of the circadian-controlled genes of each species. It has been observed that patterns of gene expression may drastically change as result of hybridization and increased ploidy in *Brassica napus* and *Arabidopsis thaliana* [51]–[53]. Ploidy and hybridity effects in the circadian clock have been associated with increase of vigour in Arabidopsis [52], [53].

Comparison among similar datasets from other species showed that there is some overlap between circadian transcripts among different species. However, when the phase of these genes where compared, the coefficient of determination varied accordingly to the phylogenetic distance between the species, showing that the control of the plant circadian clock over its outputs is a process that is constantly changing, possibly to increase the resonance of the plant's rhythms with the environmental rhythms.

Based on the available transcripts and available sugarcane sequences, the sugarcane circadian clock has similar components to the circadian clock of Arabidopsis but it also has differences: i) only one of the MYB-like transcription factor *AtCCA1*/*AtLHY* has been identified so far; ii) only four *PSEUDO RESPONSE REGULATOR* were found, while Arabidopsis has five; iv) *ScPRR3*, *ScPRR59* and *ScPRR7* peak at the same time of the day and not in slightly different phases; iv) *ScFIO*, *ScZTL*-1 and *ScZTL*-2 are rhythmic; and v) no sequences similar to *AtELF4* and *AtCHE* could be found (Figure 3 and 4). *AtELF4*, *AtLUX* and *AtELF3* have been shown to form the evening complex, which is essential to the maintenance of the circadian clock function [3], [6]. These differences, however, did not impair the sugarcane circadian clock, which suggests that the interactions between the components of the sugarcane circadian clock may also show differences from Arabidopsis. It is worth noting that the presence of common components of the core oscillator between sugarcane and Arabidopsis does not necessarily mean that they will have the same function. It is also possible that there are more differences in the sugarcane circadian clock as we did not try to identify elements of the circadian clock that are present in sugarcane, or grasses, but not in Arabidopsis.

One important question is how the circadian clock relays time information to other pathways. We have found 111 transcription factors, of a total of 356 identified in the SUCEST database, regulated by the circadian clock (31.2%), including *ScCCA1* and *ScTOC1* [54]. Moreover, a large number of probes associated with histone regulation, spliceosome function, RNA degradation and transport are circadian clock regulated (Table 5). These are mechanisms known to be involved in the control of transcript levels by the circadian clock [14], [15], [35], [55]–[59]. Another important regulatory mechanism is the expression of natural antisense transcripts (NATs). NATs have been shown to regulate their cognate transcript levels through gene methylation, direct control of transcription, alternative splicing and transcript stability [30]–[33]. Widespread antisense transcription has been described in Arabidopsis [35], [60]. In this work, not only have we found AS that were co-regulated with their SS cognates, but AS that had expression levels that did not correlated with their SS cognates, as well (Figure 3). The circadian AS time courses had a tendency to peak towards subjective dawn, while the circadian SS time courses had a tendency to peak towards the subjective middle of the day, a difference that was also observed using tiling arrays in Arabidopsis [35]. AS/SS pairs have been associated with gene plasticity and responsiveness to stimuli, which could also be a strategy to increase the amplitude of circadian rhythms [60]. Most of the circadian AS time courses identified (68%) did not have the time courses of their SS cognate considered circadian. These circadian AS may regulate the translation activity of their cognate SS in order to confer circadian behaviour in the protein level [61]. Some AS/SS pairs show small changes in phase, such as violaxanthin de-epoxidase (SCVPHR1095C07.g) and glucose 1-phosphate adenylyltransferase (SCVPCL6061A06.g) (Figure 3E and F). It is possible that these AS act controlling the expression of their SS cognate, helping to regulate the timing of their expression [31], [32] .

The sugarcane circadian transcriptome has shown some properties found in similar works, such as the clustering of transcripts with similar function, the anticipation of daily environmental changes and the temporal compartmentation of metabolic processes. Clustering can be observed in the light harvesting reactions of photosynthesis, in which probes associated with light harvesting complex and photosystem I cluster very tightly between ZT4 and ZT6, when sunlight is usually at its peak (Figure 6). Curiously, probes for synthesis of pigments precede the expression of pigment binding proteins. On the other hand, probes associated with the carbon fixing part of photosynthesis, including RuBisCo activase, tended to cluster between ZT22 and ZT2, in the beginning of the subjective day (Figure 3).

**Figure 9:**
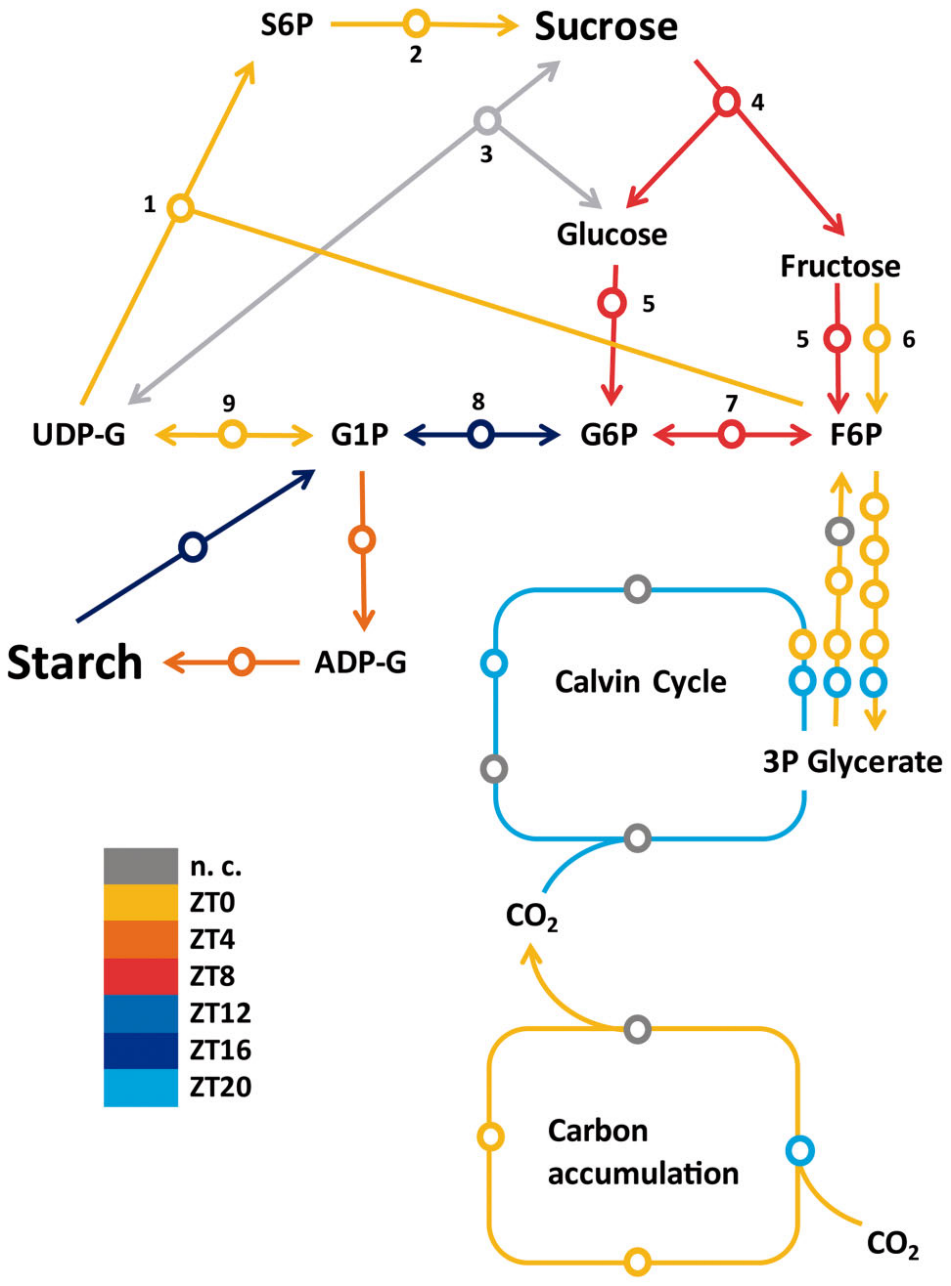
Temporal coordination of probes associated with sucrose and starch metabolism pathways. The time of peak of rhythms in transcript levels of genes associated with sucrose and starch metabolism was identified in a schematic metabolic pathway. Each circle corresponds to a specific gene model. Metabolic pathways were colored according to the median of the phase of their constitutive genes. The time of peak of probes associated with starch and sucrose synthesis pathways was between ZT20 and ZT4, while the time of the peak of probes associate to sucrose and starch degradation was between ZT8 to ZT16. Genes that were not circadian (n. c.) were in gray. Rhythmic with a time of peak at ZT0 are in yellow, ZT4 in dark orange, ZT8 in red, ZT12 in blue, ZT16 in dark blue and ZT20 in light blue. Enzymes for sucrose synthesis are: (**1**) sucrose-phosphate synthase; (**2**) sucrose phosphatase; (**3**) sucrose synthase; (**4**) neutral invertase; (**5**) hexokinase; (**6**) fructokinase; (**7**) glucose-6-phosphate isomerase; (**8**) phosphoglucomutase; (**9**) UDP-glucose pyrophosphorylase [75]. Abbreviations: S6P – sucrose 6-phosphate; UDP-G - UDP-glucose; G1P – glucose 1-phosphate; G6P – glucose 6-phosphate; F6P – fructose 6-phosphate; ADP-G – ADP-glucose; 3P glycerate – 3-phospho glycerate.

Probes for sucrose and starch metabolism are co-ordinated during the day following the timing of the carbon fixing pathways (Figure 7). There is a strong association between the peaks of probes associated with sucrose synthesis, including sucrose phosphatase synthase (SPP), with the peaks of transcript for carbon fixation and the upper portion of glycolysis/gluconeogenesis, indicating that one of the destinations of the newly fixed carbon is sucrose (Figure 9). Probes for starch synthesis cluster a few hours later. Less predominantly, two probes for sucrose degradation, including a neutral invertase (NI), cluster close to the beginning of the subjective night, around ZT8. As with sucrose and starch synthesis, starch degradation starts a few hours later. It has been shown that both the levels of probes associated with starch metabolism and their respective enzymatic activities cycle during the day but their protein levels remain constant [62], [63]. Variations of transcript levels may be important to maintain the levels of enzyme activity instead of the total amount of the enzymes. Similarly, pigment concentration in the leaves does not vary during the day (Figure S6). However, rhythms in the transcripts associated with photosynthetic pigment synthesis may follow rhythms in their damage due to photosynthetic activity, which would help maintain their concentration constant. Indeed, plants that have a circadian clock period that matches the period of environmental rhythms accumulate more chlorophyll than plants that are arrhythmic or have mismatched circadian clocks [2].

Our results suggest that sugarcane has distinct metabolic profiles during the subjective day and the subjective night. It is possible that this is a way to optimize energy and carbon allocation for two energy intensive tasks: carbon assimilation and genetic processing [64]. During the subjective day, there was dominance of probes associated with photosynthesis-related processes such as carbon fixation and carbohydrate metabolism. During the subjective night, there was dominance of probes associated with genetic processing, such as DNA replication, histone regulation and RNA polymerase, ribosome and protein synthesis. Transcripts for amino acid synthesis and sucrose, starch and lipid degradation also peak during the subjective night, providing energy and substrates for these processes during this period (Figure S4).

Not only does the circadian clock regulate a major part of the sugarcane metabolism, but its signalling pathways as well. The modulation of a signalling pathway by the circadian clock is called gating. We have found that a large proportion of probes of auxin and ABA signalling components were regulated by the circadian clock. In Arabidopsis, most transcripts of auxin signalling peaked during the subjective day, while the peak response to exogenous auxin was at subjective dawn [39], [65] (Table 5). ABA had a peak of response in the middle to the end of the subjective day [66]–[68]. In contrast, sugarcane may have two phases of auxin and ABA response (Figure 8). This suggests that the polyploidy in sugarcane have led to sub-functionalization, either at expression or at functional level, and even to neofunctionalization [69], [70]. It is possible that different parts of the sugarcane leaf are sensitive to these hormones in different times of the day. Another explanation is that sugarcane activates auxin and ABA signalling daily as a basal mechanism, synthesizing and transporting these hormones at a specific time of the day but also has other times of the day where it is more sensitive to acute auxin and ABA synthesis in response to stresses like drought, cold or wounding.

## Conclusions

Commercial sugarcane is the result of many rounds of hybridization and selection that resulted in a hybrid genome that contains variable numbers of *S. officinarum* and *S. spontaneum* genes. Despite the intense selection for the most productive individuals, the circadian clock is still capable of generating self-sustained rhythms. The circadian clock has been shown to confer survival advantages, as it allows for the anticipation of rhythmic environmental events, the temporal compartmentation of metabolic processes, the optimization of starch accumulation, and the gating of environmental signals [1], [2], [71]. It is possible that those traits allowed the commercial sugarcane become the important crop it is today.

## Materials and Methods

### Plant growth condition and harvesting

The commercial variety of sugarcane RB855453 (*Saccharum* hybrid), propagated from stem cuttings, was grown in a 1∶2 compost∶sand mix under a 12 h light (100 µmol photons m^−2^ s^−1^)/12 h dark photoperiod at 25±2°C for three months. Plants were transferred to continuous light in the beginning of the experiment. Twenty-four hours later, all the leaves from 9 different plants were harvested every 4 h for 48 h in a total of 12 time-points (between 48 h and 68 h under constant light) and frozen in liquid nitrogen. The plants were separated into three groups: two for each technical replicate of the oligoarrays and one for validation using RT-qPCR. There was no visual evidence of chlorosis in sugarcane leaves after 72 h under constant light conditions. There was also no significant difference in the levels of chlorophyll a, chlorophyll b and carotenoids levels throughout the experiment (One-way ANOVA; Figure S6).

### Oligoarray hybridizations

Total RNA extraction, labelling and hybridization were performed as described [29]. Briefly, frozen samples were pulverized, and then total RNA was extracted using Trizol (Invitrogen), treated with DNase I (Life Technologies) and purified with RNeasy® Mini Kit (Qiagen). Sample labelling was done following the Two-Colour Microarray-Based Gene Expression Analysis Protocol (Quick Amp Labelling) and hybridization was performed in a custom 4×44 k oligoarray (Agilent) using a equimolar pool of all samples as a reference. The oligoarray was designed using an in house pipeline that selected specific sequences from 14,521 Sugarcane Assembled Sequences (SAS) from the Sugarcane EST Project (SUCEST). All the SAS from SUCEST that had specific 60-mer probes that fit the quality controls were represented in the array. The quality controls used for the60-mer probe were: 1) the G% must be less than 50%; 2) CG content must be between 35% and 55%; 3) melting temperature must be between 68°C and 76°C; 4) homopolymers must be smaller than 6 bp. Probe specificity was assayed using BLASTN between the selected probes and the SUCEST. The first hit must had 100% identity with the target SAS. The following hit had a score bit lower than 42.1 and coverage of 35% or less, without gaps [29]. A total of 14,521 probes that hybridize to sense transcripts and 7,380 probes that hybridize to antisense transcripts were used in duplicate in the oligoarray. The arrays were scanned with a GenePix 4000B scanner (Molecular Devices, Sunnyvale, CA, USA) using the suggested Agilent Scan Settings. Two hybridizations were made for each time-point using independently prepared samples and dye swaps. The arrays were validated with RT-qPCR using a third set of samples, as described previously [29]. We have tested eight transcripts considered rhythmic and one considered arrhythmic. All these transcripts had similar time courses either using oligoarrays or RT-qPCR. However, the arrhythmic time course did not show a significant correlation using Spearman's rank correlation coefficient (ρ) (Figure S3). Of the 108 individual points, 88% were validated. The complete dataset can be found at the Gene Expression Omnibus public database (GEO) under the accession number: GSE42725.

### Data analysis

#### Data extraction and normalization

Data was extracted using Feature Extraction software version 9.5.3.1 (Agilent Technologies) using the protocol GE2-v5_95_Feb07. A background signal correction and a non-linear LOWESS normalization were applied to each dataset [29]. Signals that were distinguishable from the local background signal were used as an indication that the correspondent transcript was expressed and only transcripts that were considered expressed in 10 of the 12 time-points were further analysed. The time course for each transcript considered expressed was normalized by Z-score [72].

#### Identification of rhythmic transcripts

Each time course was analysed by three different algorithms to identify rhythmic behaviour: COSOPT, Fisher's G-test and JTK_CYCLE. Only time courses that were considered rhythmic by two or more algorithms were considered circadian. Both COSOPT and JTK_CYCLE measure how close a time course fits to a series of cosine waves with different phases and period lengths between 20 h and 28 h [37], [38]. Fisher's G-test is an algorithm that detects rhythmic time courses using Fourier transform [36]. The period was estimated using COSOPT and the phase was estimated using JTK_CYCLE.

#### Term enrichment analysis

Each SAS in the oligoarray was automatically and manually annotated and categorized in thirty functional categories. Term enrichment analysis was done using the GeneMerge tool [73], which compares the frequency of each category among the circadian SAS and compares with the frequency of each category in all the SAS contained in the whole array [29].

#### Identification of ortholog clusters

Ortholog clusters were generated using InParanoid and MultiParanoid [41], [42]. InParanoid was used to make pairwise proteome comparisons of 39,021 sugarcane proteins predicted from 43,141 SAS [74], sorghum (36,338 proteins), maize (106,046 proteins), rice (51,258 proteins) and Arabidopsis (35,386 proteins). This algorithm uses a hexanucleotide-based Markov chain in order to model coding regions even when they have sequencing errors that induce frame shifts or indels [42]. MultiParanoid was used to create 18,611 multi-species ortholog clusters through the aggregation of results from InParanoid comparisons [42]. We used: confidence score > = 0.05, score cutoff > = 40 bits, sequence overlap cutoff > = 0.5, group merging cutoff > = 0.5, and the BLOSUM80 matrix, as parameters in this analysis.

#### Pairwise comparison between arrays

In order to make pairwise comparisons between two arrays, we have converted each annotated probe to its respective ortholog cluster. Only the ortholog clusters that were found in both arrays were used. If an ortholog cluster contained more than one probe in the array, it was still counted once. In these cases, if one probe was considered circadian, the whole ortholog cluster was labelled as circadian. Then, the list of common orthologous clusters was compared for common circadian ortholog clusters. The ortholog clusters considered circadian in both arrays were separated for phase analysis. The phase of each ortholog cluster was determined by the median of all circadian probes assigned for that ortholog cluster. The difference between a pair of phases was always minimized. For example, if a pair of ortholog clusters have phases ZT2 and ZT22, the comparison was made between ZT26 and ZT22.

## Supporting Information

Figure S1 **Identification of rhythmic probes in other datasets.** (**A**) A maize dataset [18], (**B**) a rice dataset [17]and (**C–D**) two Arabidopsis circadian datasets [20], [39] were reanalysed using our analysis pipeline. Venn diagrams showing the number of transcripts in each dataset that were considered rhythmic by three algorithms: JTK_CYCLE, COSOPT and Fisher's G-test.
(TIF)

Figure S2 **More than one sugarcane assembled sequences (SAS) may align to a same gene model.** The SbTOC1 CDS (1,732 bp) was blasted against the Sugarcane EST database and two different SAS were selected: SCEPLB1042B08.g (381 bp) and SCCCSB1002H04.g (1,256 bp).
(TIF)

Figure S3 **Real-time PCR validation of array time courses.** Z-score normalized expression levels from the arrays (darker colour) and from real-time PCR (lighter colour) for (**A**) *ScCCA1* (SCCCLR1048E10.g), (**B**) *ScTOC1* (SCCCSB1002H04.g and SCEPLB1042B08.g), (**C**) *ScGI* (SCJFAD1014B07.b), (**D**) *ScPRR*3 (SCACLR1057G02.g), (**E**) *ScPRR*59 (SCCCLR1077F09.g), (**F**) *ScPRR7* (SCACLR1057C07.g), (**G**) *ScPP2C* (SCEPRZ1010E06.g), (**H**) *ScPSI* (SCQGLR2025B12.g), and (**I**) *ScCAB2* (SCUTST3086G11.g). Spearman's rank correlation coefficient (ρ) between the array and real-time PCR time courses is shown. Significant correlations were marked with a * (p>0.56 or p<−0.56). White boxes represent periods of subjective day and light grey boxes represent periods of subjective night.
(TIF)

Figure S4 **Rhythmic probes associated with several pathways.** Z-score normalized time courses of rhythmic probes for transcripts associated with the photosynthetic pathway were separated into (**A–B**) amino acid metabolism; (**C–D**) lipid metabolism; (**E**) nitrogen metabolism; and (**F**) flowering regulation. Lines in different colours indicate transcripts with contrasting phases. White boxes represent periods of subjective day and light grey boxes represent periods of subjective night.
(TIF)

Figure S5 **Rhythmic probes associated with several hormone signalling pathways.** Z-score normalized time courses of rhythmic probes for transcripts associated with the photosynthetic pathway were separated into (**A**) brassinosteroids signalling (green) and (**B**) ethylene signalling (red). Lines in different colours indicate transcripts with contrasting phases. White boxes represent periods of subjective day and light grey boxes represent periods of subjective night.
(TIF)

Figure S6 **Levels of photosynthetic pigments remained constant in constant light.** Leaf pigments were extracted using chilled 80% acetone and measured using a spectrophotometer. Briefly, 100 mg of frozen ground leaf tissue was homogenized in 10 ml 80% acetone for 72 h, protected from light at 4°C. Samples were measured in sealed 96-well plates, to avoid acetone evaporation, using a plate spectrophotometer (BMG Labtech). Absorbance was measured at 480 nm (A_488_), 645 nm (A_645_) and 663 nm (A_663_). Pigments concentrations were calculated using the Arnon equations and then normalized for 80% acetone volume used and the amount of tissue used in weight.
(TIF)

Table S1 **Ortholog clusters validation using enzymes involved in sucrose synthesis and degradation.** The ortholog clusters generated using InParanoid and MultiParanoid were compared with genes annotated manually. The number of false positives is the number of sequences that were present in an ortholog cluster but did not match the other genes. The number of false negatives is the number of sequences that were manually annotated as an enzyme but were not present in any ortholog clusters.
(DOCX)

Table S2 **Term enrichment of rhythmic probes in the sense direction.**
(DOCX)

Table S3 **Term enrichment of rhythmic probes in the antisense direction.**
(DOCX)
