# Supplementary figures and images for: Circadian Rhythms of Sense and Antisense Transcription in Sugarcane, a Highly Polyploid Crop
Source: PLoS One. 2013 Aug 6;8(8):e71847. doi: 10.1371/journal.pone.0071847 (PMC3735537; doi:10.1371/journal.pone.0071847)

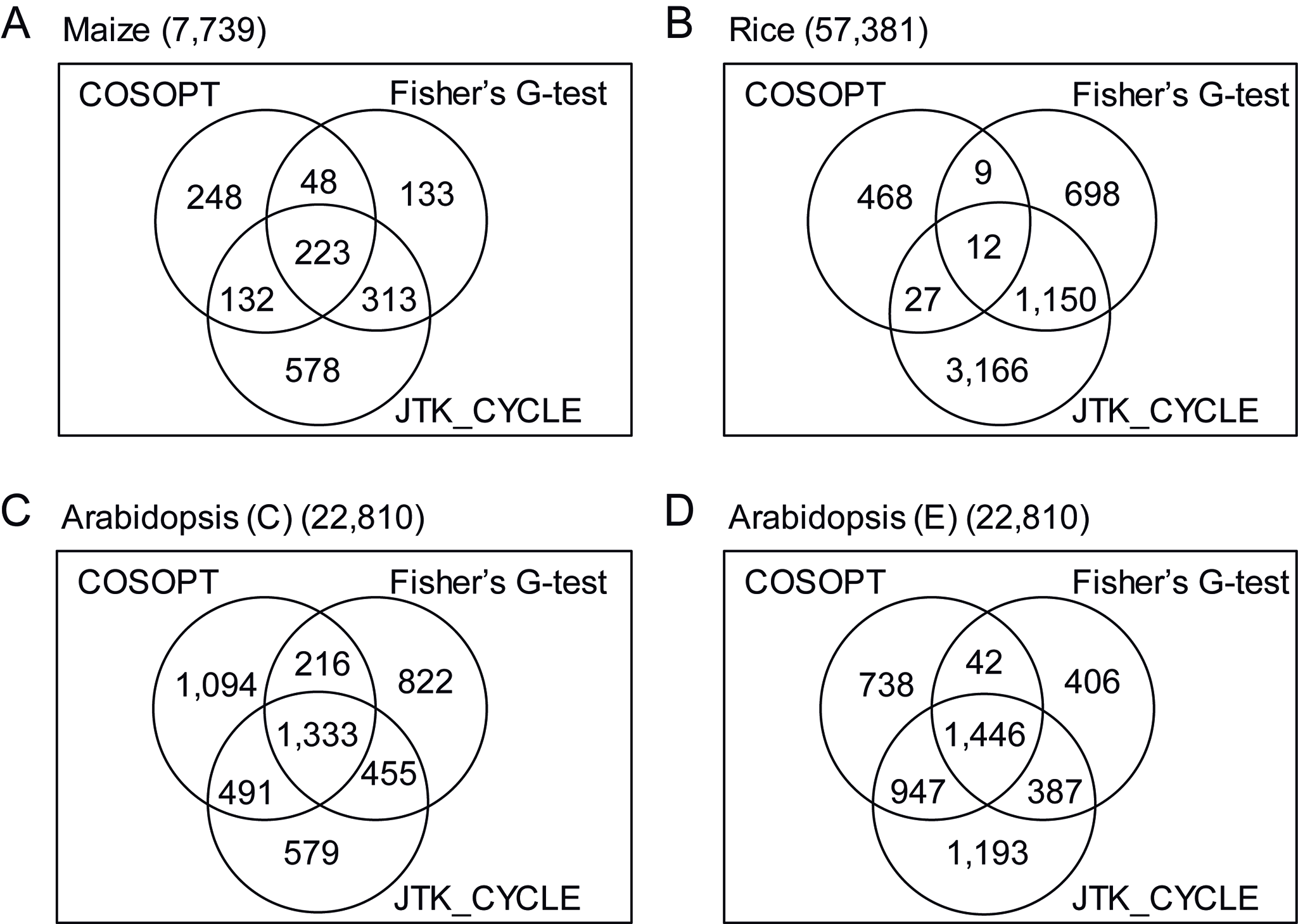

Supplement: Figure S1 — Identification of rhythmic probes in other datasets. (A) A maize dataset [18], (B) a rice dataset [17]and (C–D) two Arabidopsis circadian datasets [20], [39] were reanalysed using our analysis pipeline. Venn diagrams showing the number of transcripts in each dataset that were considered rhythmic by three algorithms: JTK_CYCLE, COSOPT and Fisher's G-test. (TIF) [file pone.0071847.s001.tif]

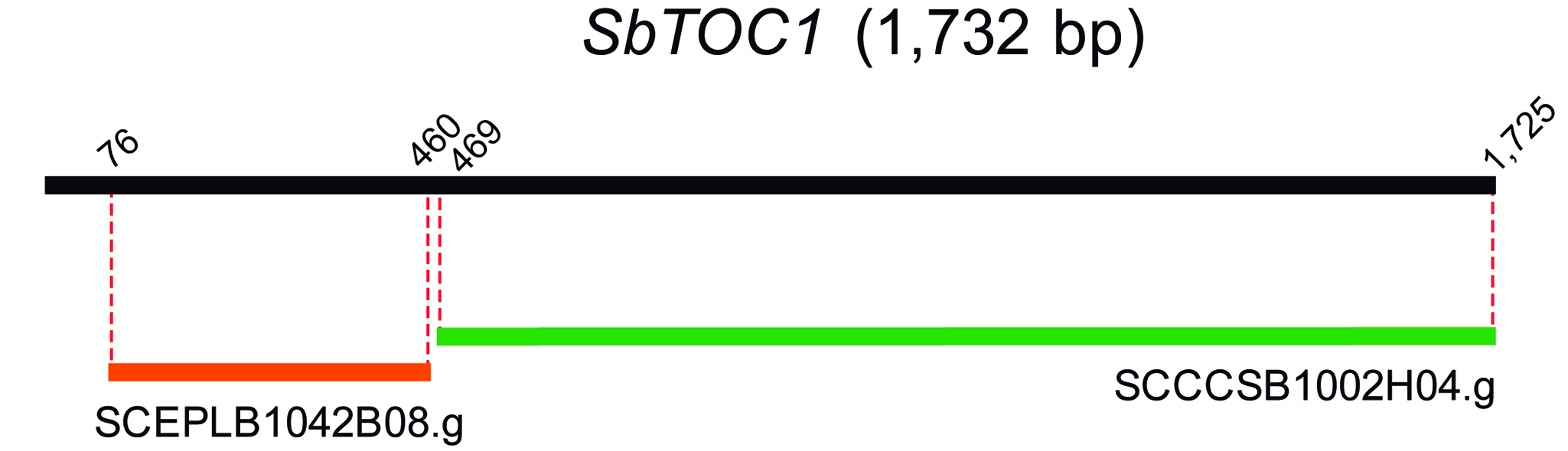

Supplement: Figure S2 — More than one sugarcane assembled sequences (SAS) may align to a same gene model. The SbTOC1 CDS (1,732 bp) was blasted against the Sugarcane EST database and two different SAS were selected: SCEPLB1042B08.g (381 bp) and SCCCSB1002H04.g (1,256 bp). (TIF) [file pone.0071847.s002.tif]

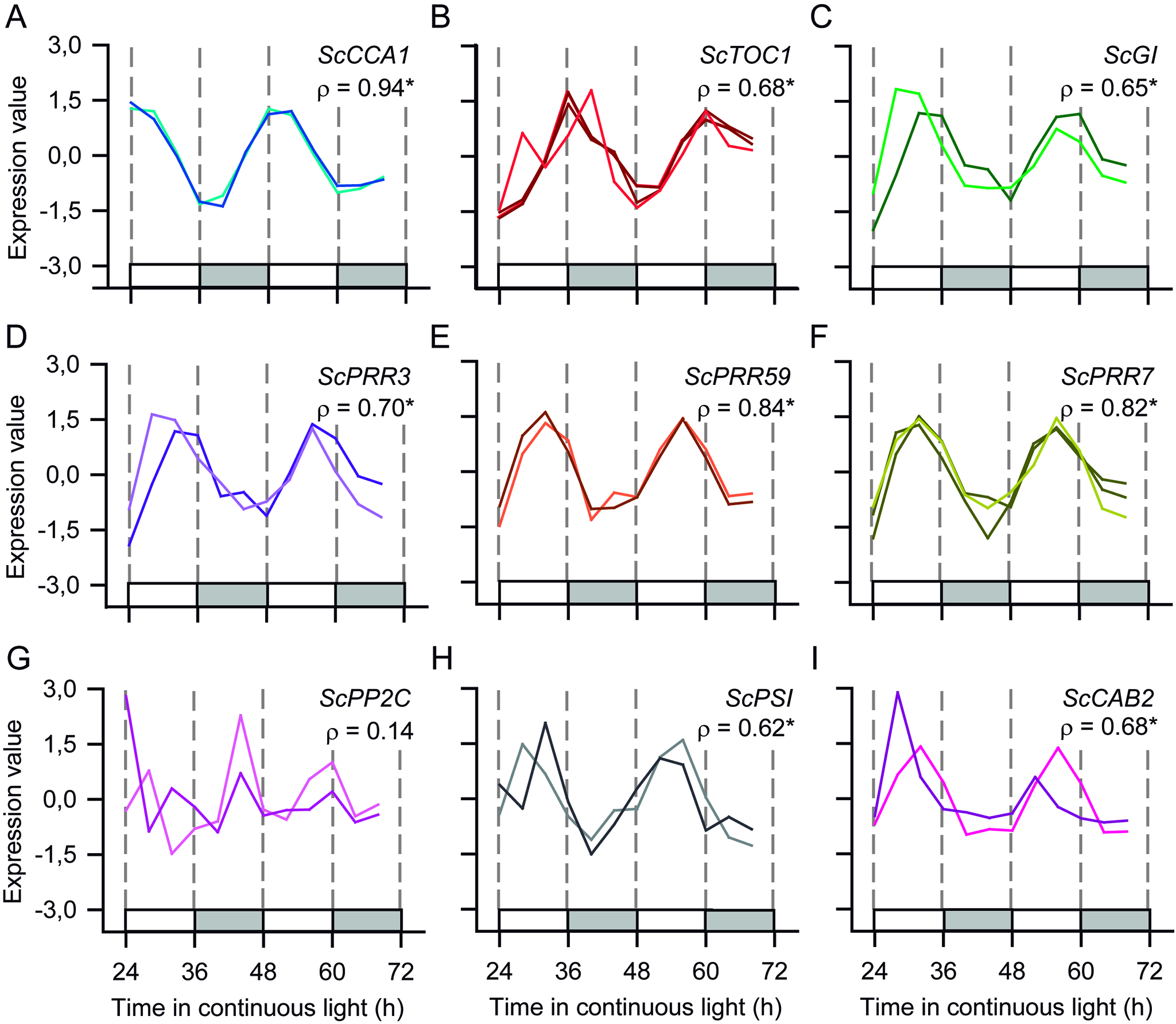

Supplement: Figure S3 — Real-time PCR validation of array time courses. Z-score normalized expression levels from the arrays (darker colour) and from real-time PCR (lighter colour) for (A) ScCCA1 (SCCCLR1048E10.g), (B) ScTOC1 (SCCCSB1002H04.g and SCEPLB1042B08.g), (C) ScGI (SCJFAD1014B07.b), (D) ScPRR3 (SCACLR1057G02.g), (E) ScPRR59 (SCCCLR1077F09.g), (F) ScPRR7 (SCACLR1057C07.g), (G) ScPP2C (SCEPRZ1010E06.g), (H) ScPSI (SCQGLR2025B12.g), and (I) ScCAB2 (SCUTST3086G11.g). Spearman's rank correlation coefficient (ρ) between the array and real-time PCR time courses is shown. Significant correlations were marked with a * (p>0.56 or p<−0.56). White boxes represent periods of subjective day and light grey boxes represent periods of subjective night. (TIF) [file pone.0071847.s003.tif]

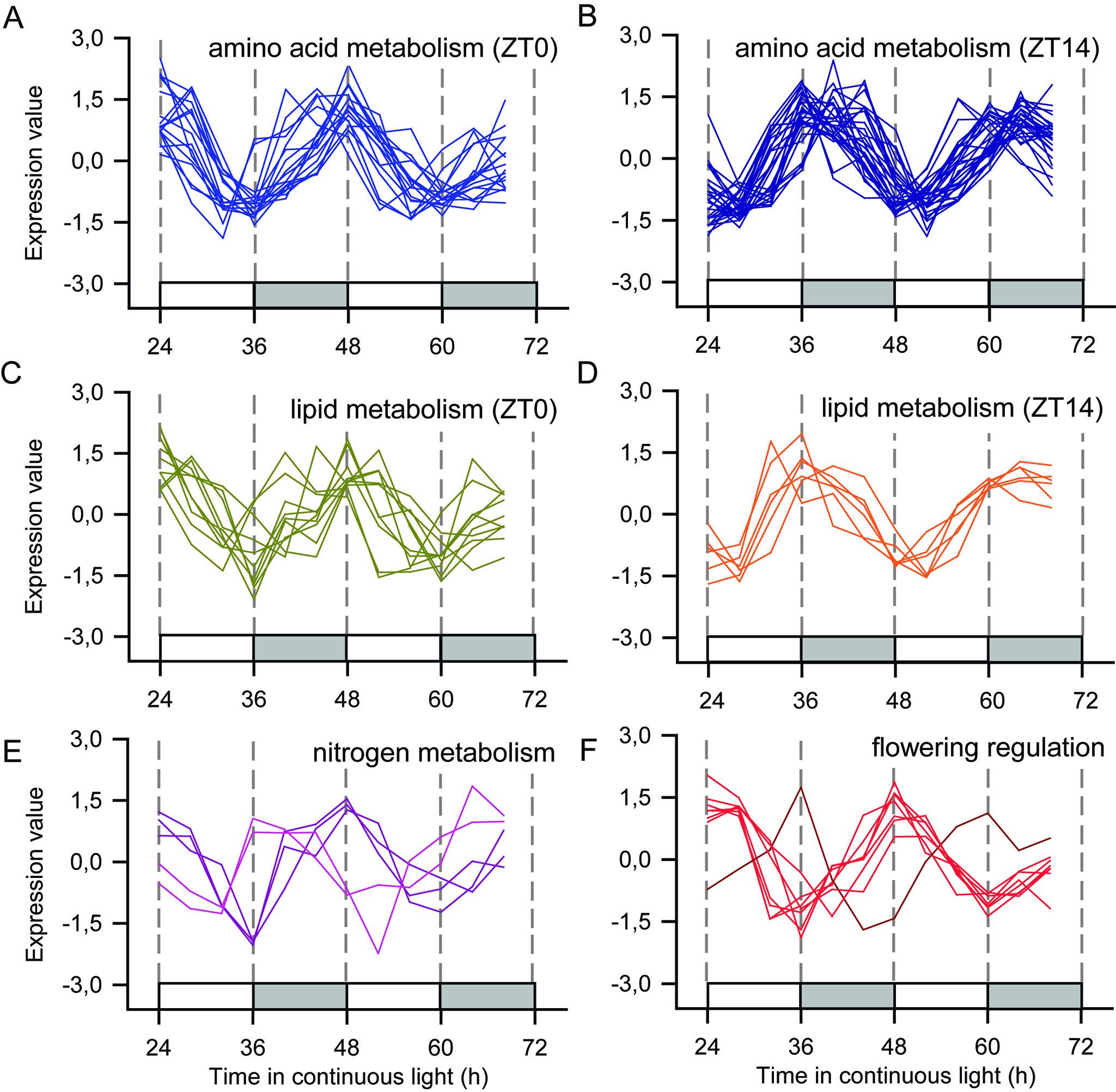

Supplement: Figure S4 — Rhythmic probes associated with several pathways. Z-score normalized time courses of rhythmic probes for transcripts associated with the photosynthetic pathway were separated into (A–B) amino acid metabolism; (C–D) lipid metabolism; (E) nitrogen metabolism; and (F) flowering regulation. Lines in different colours indicate transcripts with contrasting phases. White boxes represent periods of subjective day and light grey boxes represent periods of subjective night. (TIF) [file pone.0071847.s004.tif]

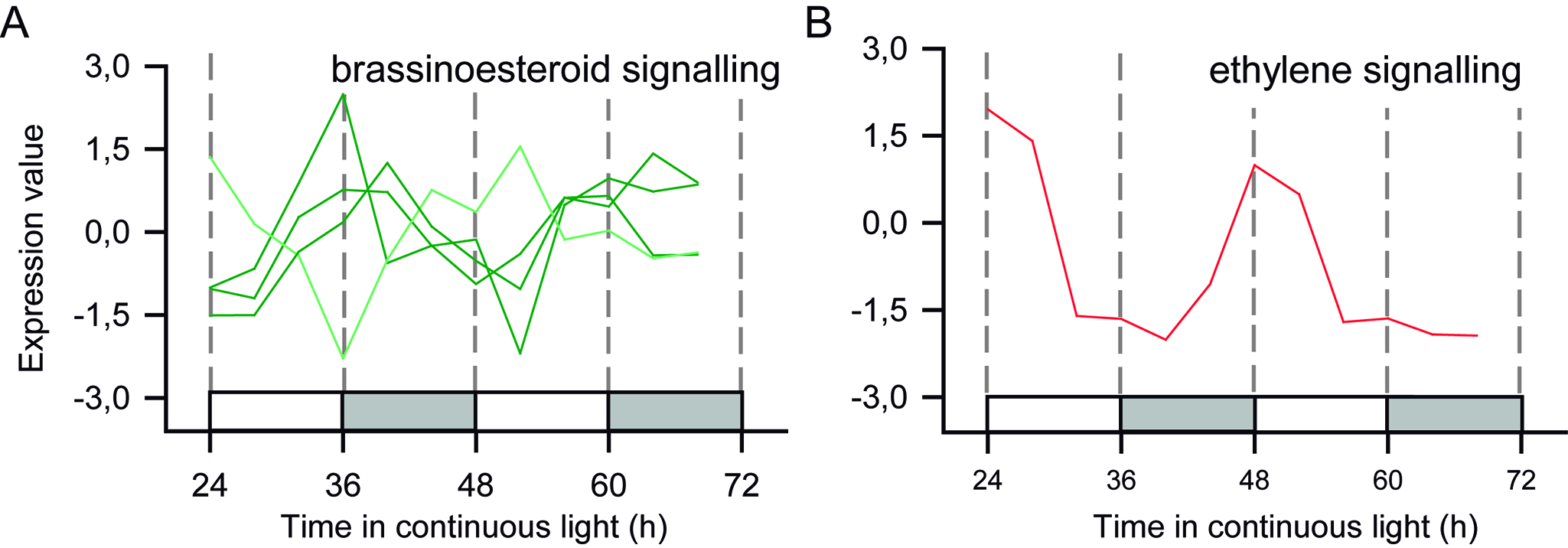

Supplement: Figure S5 — Rhythmic probes associated with several hormone signalling pathways. Z-score normalized time courses of rhythmic probes for transcripts associated with the photosynthetic pathway were separated into (A) brassinosteroids signalling (green) and (B) ethylene signalling (red). Lines in different colours indicate transcripts with contrasting phases. White boxes represent periods of subjective day and light grey boxes represent periods of subjective night. (TIF) [file pone.0071847.s005.tif]

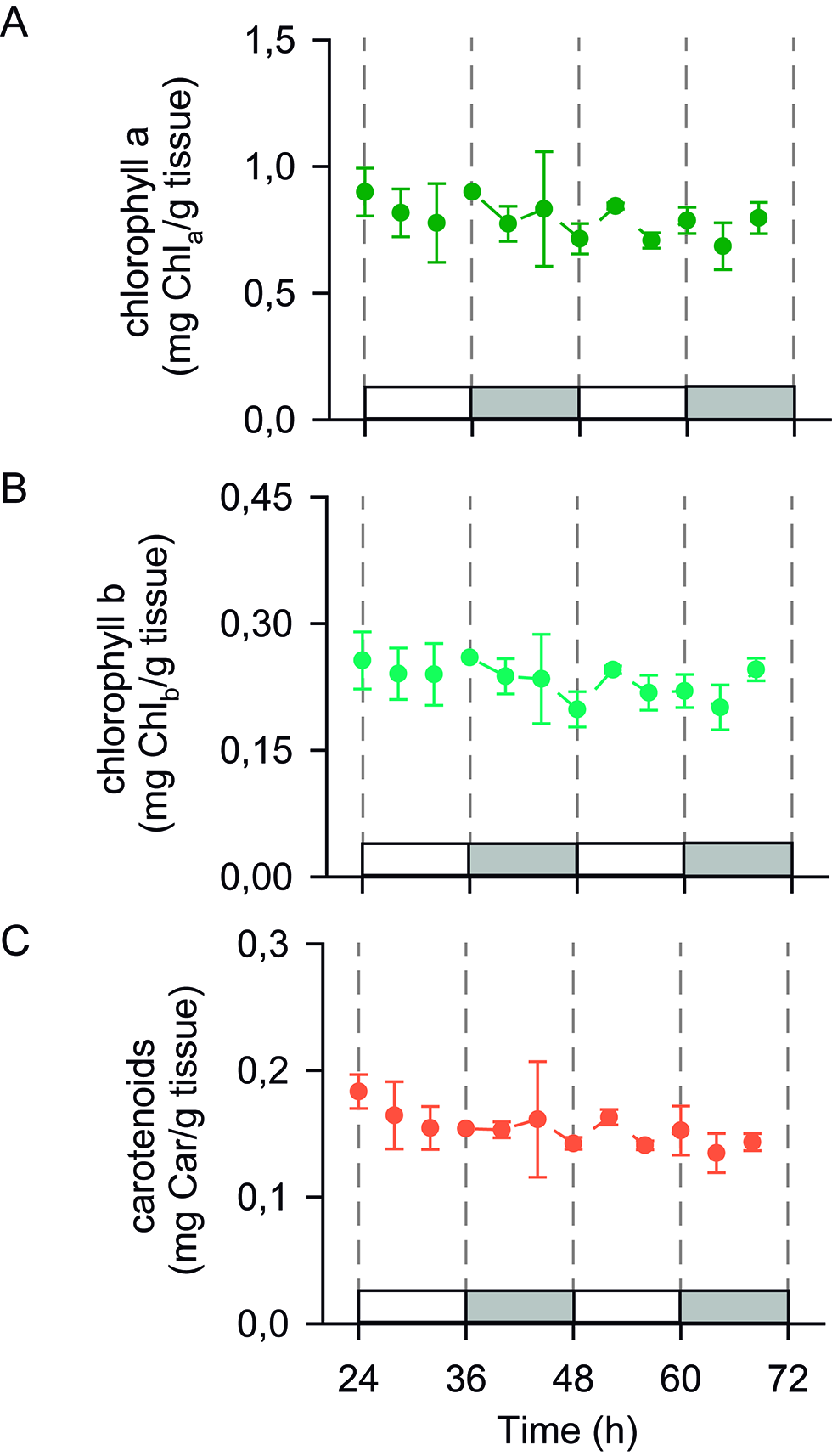

Supplement: Figure S6 — Levels of photosynthetic pigments remained constant in constant light. Leaf pigments were extracted using chilled 80% acetone and measured using a spectrophotometer. Briefly, 100 mg of frozen ground leaf tissue was homogenized in 10 ml 80% acetone for 72 h, protected from light at 4°C. Samples were measured in sealed 96-well plates, to avoid acetone evaporation, using a plate spectrophotometer (BMG Labtech). Absorbance was measured at 480 nm (A488), 645 nm (A645) and 663 nm (A663). Pigments concentrations were calculated using the Arnon equations and then normalized for 80% acetone volume used and the amount of tissue used in weight. (TIF) [file pone.0071847.s006.tif]
