# Supplementary material for: Circadian Rhythms of Sense and Antisense Transcription in Sugarcane, a Highly Polyploid Crop
Source: PLoS One. 2013 Aug 6;8(8):e71847. doi: 10.1371/journal.pone.0071847 (PMC3735537; doi:10.1371/journal.pone.0071847)
Supplement: Table S1 — Ortholog clusters validation using enzymes involved in sucrose synthesis and degradation. The ortholog clusters generated using InParanoid and MultiParanoid were compared with genes annotated manually. The number of false positives is the number of sequences that were present in an ortholog cluster but did not match the other genes. The number of false negatives is the number of sequences that were manually annotated as an enzyme but were not present in any ortholog clusters. (DOCX) [file pone.0071847.s007.docx]

**Table S1**

|  |  | Sugarcane | | Maize | | Rice | | Arabidopsis | |
| --- | --- | --- | --- | --- | --- | --- | --- | --- | --- |
| Enzyme | **clusters** | **clusters (genes)** | **false positives/ negatives** | **clusters (genes)** | **false positives/ negatives** | **clusters (genes)** | **false positives/ negatives** | **clusters (genes)** | **false positives/ negatives** |
| sucrose-phosphate synthase | 5 | 3 (9) | 0/6 | 5 (7) | 0/0 | 4 (5) | 0/1 | 3 (3) | 0/0 |
| sucrose phosphatase | 1 | 1 (2) | 0/1 | 1 (2) | 0/0 | 1 (3) | 0/0 | 1 (2) | 1/0 |
| UDPG pyrophosphorylase | 1 | 1 (4) | 0/3 | 1 (2) | 0/0 | 1 (4) | 0/1 | 1 (2) | 0/0 |
| fructokinase | 3 | 3 (5) | 0/1 | 3 (5) | 0/0 | 3 (3) | 0/0 | 3 (7) | 0/1 |
| sucrose synthase | 2 | 2 (13) | 0/9 | 2 (7) | 0/1 | 1 (7) | 0/2 | 1 (6) | 0/2 |
| neutral invertase | 3 | 2 (2) | 0/0 | 3 (6) | 0/1 | 3 (3) | 0/0 | 1 (4) | 0/1 |
| hexokinase | 7 | 4 (13) | 0/7 | 7 (10) | 0/0 | 7 (10) | 0/0 | 1 (6) | 0/2 |
| glucose-6-phosphate isomerase | 3 | 2 (6) | 1/4 | 3 (4) | 0/1 | 2 (3) | 0/0 | 2 (2) | 0/0 |
| phosphoglucomutase | 3 | 2 (2) | 0/0 | 3 (4) | 0/0 | 2 (2) | 0/0 | 2 (3) | 0/0 |
| Total | **28** | **20 (56)** | **1/31** | **28 (47)** | **0/3** | **24 (40)** | **0/4** | **15 (35)** | **1/6** |
