# Supplementary material for: Circadian Rhythms of Sense and Antisense Transcription in Sugarcane, a Highly Polyploid Crop
Source: PLoS One. 2013 Aug 6;8(8):e71847. doi: 10.1371/journal.pone.0071847 (PMC3735537; doi:10.1371/journal.pone.0071847)
Supplement: Table S2 — Term enrichment of rhythmic probes in the sense direction. (DOCX) [file pone.0071847.s008.docx]

**Table S2**

| Functional Category | e-score |
| --- | --- |
| Unknown | 1.25E-26 |
| Protein metabolism | 1.16E-13 |
| Others | 2.17E-12 |
| RNA metabolism | 1.96E-11 |
| Transporters | 1.08E-06 |
| Amino acid and nitrogen metabolism | 1.86E-06 |
| Light harvesting | 1.50E-05 |
| DNA metabolism | 4.83E-05 |
| Porphyrin and chlorophyll metabolism | 3.20E-04 |
| Transcription regulation | 3.68E-04 |
| Carbohydrate metabolism | 8.76E-04 |
| Signal Transduction | 5.68E-02 |
| Nucleotide metabolism | 7.58E-02 |
| Maintenance of genetic material | 1.46E-01 |
| Modification and protein degradation | 2.66E-01 |
| Oxidative phosphorylation | 6.76E-01 |
