# Supplementary material for: Circadian Rhythms of Sense and Antisense Transcription in Sugarcane, a Highly Polyploid Crop
Source: PLoS One. 2013 Aug 6;8(8):e71847. doi: 10.1371/journal.pone.0071847 (PMC3735537; doi:10.1371/journal.pone.0071847)
Supplement: Table S3 — Term enrichment of rhythmic probes in the antisense direction. (DOCX) [file pone.0071847.s009.docx]

**Table S3**

| Functional Category | e-score |
| --- | --- |
| Unknown | 4.58E-09 |
| Porphyrin and chlorophyll metabolism | 1.01E-02 |
| Protein metabolism | 1.22E-01 |
| Cytoskeleton and vesicle transport | 2.47E-01 |
| Pathogen Resistance | 2.48E-01 |
| Maintenance of genetic material | 4.11E-01 |
| Light harvesting | 4.79E-01 |
